# STAID: A Self‐Refining Deep Learning Framework for Spatial Cell‐Type Deconvolution with Biologically Informed Modeling

**DOI:** 10.1002/advs.75607

**Published:** 2026-05-10

**Authors:** Jixin Liu, Shuli Sun, Zhengliang Lv, Xinyu Liu, Yihua Wang, Bingqiang Liu

**Affiliations:** ^1^ School of Mathematics Shandong University Jinan Shandong China; ^2^ Mathematical Research Center Shandong University Jinan Shandong China; ^3^ Research Center for Mathematics and Interdisciplinary Sciences (Frontiers Science Center for Nonlinear Expectations) Shandong University Qingdao China; ^4^ Breast Center The Second Hospital of Shandong University Jinan Shandong China; ^5^ State Key Laboratory of Cryptography and Digital Economy Security Shandong University Jinan Shandong China

**Keywords:** cell‐type deconvolution, graph fourier transform, pseudo‐spot refinement, spatial transcriptomics

## Abstract

Spatial transcriptomics provides high‐throughput measurement of gene expression while retaining spatial context; however, inferring accurate cell‐type compositions within individual spots remains a major challenge. Here, we present STAID, a unified framework that effectively integrates pseudo‐spot generation with deep learning training through iterative pseudo‐spot refinement and leverages graph signal processing to capture higher‐order gene‐wise relationships. By creating a self‐reinforcing cycle, STAID enables accurate spot‐level deconvolution of cell‐type compositions for spatial transcriptomics data. Comprehensive benchmarking demonstrates that STAID outperforms existing methods, accurately reconstructs cell‐type spatial distributions, and effectively resolves the cellular colocalization. In clinical breast cancer sections, STAID precisely infers tumor epithelial distributions and reveals their spatial associations with immune cells. In human embryonic limb datasets, STAID captures the ordered spatial distributions of key progenitor populations, reflecting hierarchical tissue organization and demonstrating that incorporating cell‐type composition information can enhance tissue segmentation. STAID also resolves the spatial cellular organization in Crohn's disease and reveals the characteristics of TLS‐like immune niches. Collectively, by delivering high‐resolution cell‐type distributions, STAID provides deeper insights into tissue organization and cellular heterogeneity.

## Graphical abstract

STAID is a unified deep learning framework that couples iterative pseudo‐spot refinement with neural network training through a feedback loop and exploits gene co‐expression information to model higher‐order interactions, achieving accurate and robust cell‐type deconvolution in spatial transcriptomics. By persistently generating pseudo‐spots that closely resemble real data and establishing a virtuous cycle, STAID reflects the characteristics of the target tissue compositions.

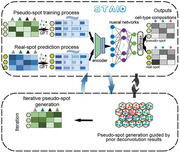

## 1 Introduction

Spatial transcriptomics (ST) has emerged as a powerful tool for profiling gene expression within intact tissue contexts [1], offering a systems‐level view of spatial transcriptional patterns [2, 3, 4], tissue architecture [5, 6, 7], and cell–cell interactions [8]. This advancement has paved the way for deeper insights into fundamental biological processes, such as neuroscience [9, 10, 11], embryonic development [12, 13, 14], tumor progression [15, 16, 17, 18], and other complex diseases [19, 20, 21, 22, 23, 24]. Several spatial transcriptomics technologies have reached single‐cell resolution [25, 26, 27], but they typically remain constrained by limited gene coverage. Some computational approaches have been proposed to enhance the data quality and utility for these spatial transcriptomics technologies [28, 29, 30, 31]. Nevertheless, most current ST technologies remain constrained by their limited spatial resolution: instead of resolving single‐cell transcriptomes, each spot typically captures a mixture of multiple cells, ranging from a few to dozens depending on the platform [32]. For example, the widely adopted 10x Genomics Visium platform employs 55 µm spots that typically capture several to dozens of cells, yet it has already generated extensive spatial transcriptomic datasets [33]. This limitation highlights the urgent need for robust cell‐type deconvolution methods that can disentangle the mixed signals in each spot, accurately estimating the proportional distributions of constituent cell types, thereby unlocking the full biological potential of ST data.

A variety of deconvolution methods for spatial transcriptomics have been developed that follow the inevitable strategy of leveraging single‐cell RNA sequencing (scRNA‐seq) data as a reference, decomposing mixed gene expression profiles into contributions from distinct cell types and thereby inferring the underlying cell‐type compositions [34, 35, 36, 37]. Among them, most intuitive strategies come from traditional probability and optimization models. For example, SpatialDWLS [38] adapts weighted least squares regression with cell‐type–specific markers and spatial constraints to efficiently estimate cell‐type proportions. Cell2location [39] employs a Bayesian framework that integrates scRNA‐seq–derived cell‐type signatures to infer cell‐type compositions [39]. RCTD [40] uses a probabilistic model to decompose ST spots into cell‐type proportions while accounting for gene expression variability. However, these approaches typically rely on explicit distributional or linear assumptions, which constrain their ability to model the nonlinear dependencies inherent in gene expression and complex cell‐type mixtures.

Deep‐learning‐based methods offer a more promising route for ST deconvolution by learning the nonlinear mapping between cell‐type compositions and pseudo‐spot gene expression generated from scRNA‐seq data. For example, DSTG [41] employs graph‐based convolutional networks to infer the composition of ST data in a semi‐supervised manner, with pseudo‐spots randomly generated as priors. STdGCN [42] integrates scRNA‐seq expression profiles with spatial information from ST data, and applies graph neural networks to deconvolve cell‐type compositions, also relying on randomly generated pseudo‐spots as priors. However, in existing methods, pseudo‐spots are generated outside the neural network pipeline, often through fully random sampling, and remain fixed before deep learning training, preventing them from being updated via gradient‐based optimization. As a result, their quality is suboptimal and their distributions diverge substantially from those of real spatial transcriptomics spots, introducing distribution shifts that undermine model performance. Besides, the lack of critical gene‐gene dependencies restricts the capture of co‐expression patterns and higher‐order interactions that may encode important biological signals necessary for accurate inference of cell‐type compositions.

Here, we present STAID, a unified deep learning framework that fundamentally rethinks the role of training data construction in spatial transcriptomics deconvolution. Unlike existing methods that rely on pre‐generated pseudo‐spots and treat data generation and model training as two independent stages, STAID tightly couples these processes within a self‐refining feedback loop. In this framework, pseudo‐spot generation is no longer a static preprocessing step, but an adaptive component that co‐evolves with model optimization. Specifically, improved pseudo‐spots enhance model learning, while the updated model in turn generates higher‐quality pseudo‐spots, forming a closed‐loop system that progressively improves model performance [43]. In addition, STAID explicitly models gene dependencies by incorporating gene co‐expression structure through graph signal processing. Rather than treating genes as independent features, we represent gene expressions of a spot as a graph signal defined on a gene co‐expression network and transform them into the frequency domain via graph Fourier transform (GFT). This formulation enables the model to capture higher‐order expression patterns [44, 45, 46, 47]. STAID further applies low‐pass filtering in the frequency domain to emphasize biologically meaningful co‐expression signals while suppressing noise, thereby improving the robustness and generalizability of cell‐type composition inference [47, 48, 49].

We comprehensively validated the performance of STAID on both simulated and real spatial transcriptomics datasets, where it consistently inferred ground‐truth cell‐type compositions across diverse testing scenarios and demonstrated spatial concordance between predicted cell types and marker gene distributions, underscoring its robustness and reliability. In addition, STAID accurately captured cell‐type colocalization patterns, highlighting their relevance for studying cell–cell interactions and tissue functional niches. In breast cancer samples, STAID accurately mapped the spatial distribution of tumor epithelial and other cell types, capturing biologically meaningful cell–cell colocalization patterns, including the spatial interactions between tumor and immune cells. In human embryonic limb datasets, STAID faithfully reconstructed the spatial distributions of progenitor populations, capturing hierarchical tissue organization and coherent spatial relationships. Furthermore, the cell‐type composition information derived by STAID effectively delineated tissue architecture during limb development. In human Crohn's disease datasets, STAID profiled spatial cell‐type distribution patterns across intestinal tissues, capturing the cellular organization of epithelial, mucosal, and vascular compartments. STAID also resolved spatial cell‐type organization and revealed immune cell–enriched, tertiary lymphoid structure (TLS)‐like niches associated with chronic inflammation. Together, these results highlight STAID's ability to accurately and robustly characterize cellular compositions and spatial organization, providing a powerful framework for dissecting tissue architecture and cellular heterogeneity across diverse biological systems.

## 2 Results

### 2.1 Overview of STAID

**FIGURE 1:**
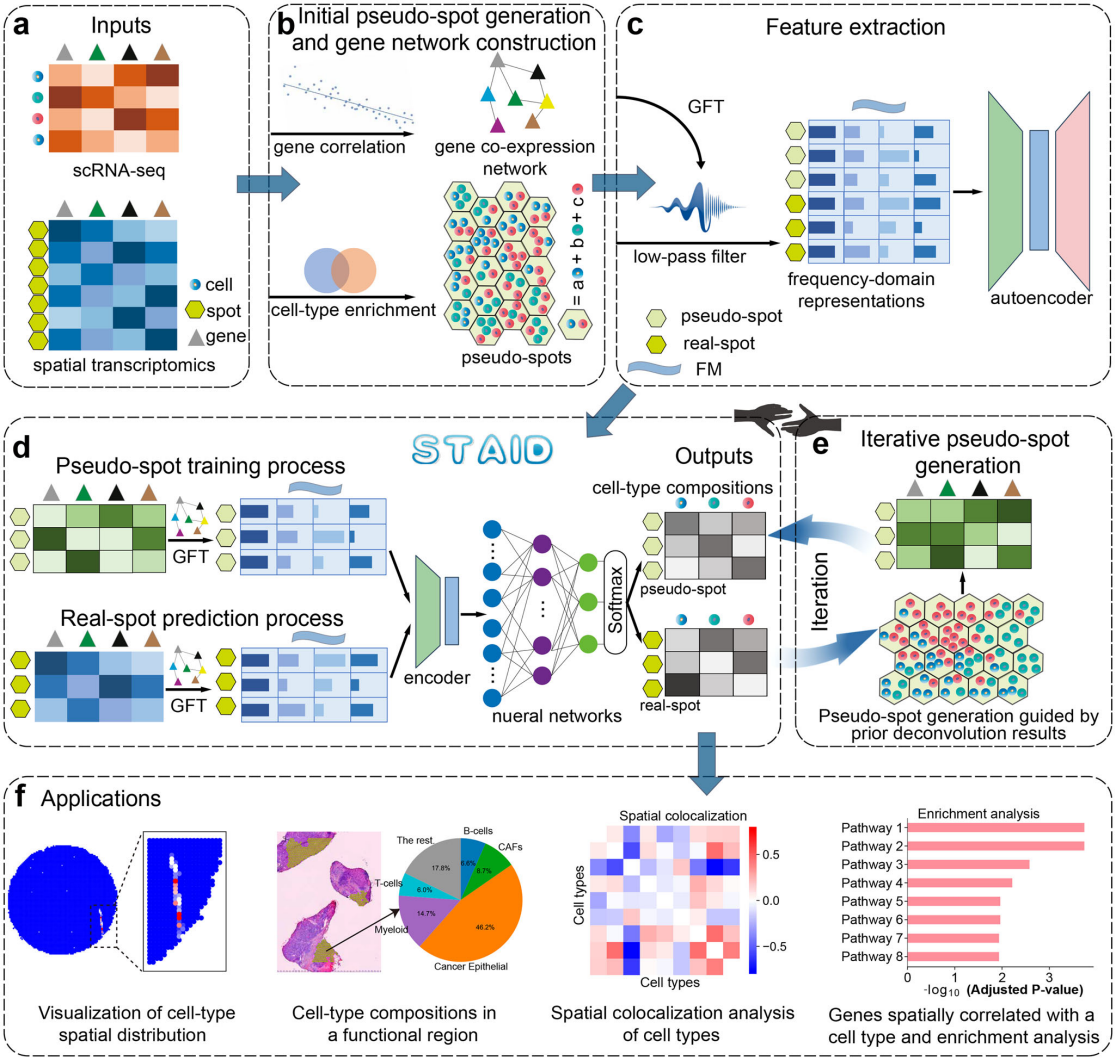
Framework of STAID. (a) Inputs include an scRNA‐seq dataset, which provides single‐cell gene expression profiles with cell‐type annotation information, and a spatial transcriptomics dataset, which contains gene expression profiles for spatial spots. (b) Unlike existing methods that generate pseudo‐spots through fully random sampling, STAID generates pseudo‐spots by sampling from the potential cell types of each real‐spot based on cell‐type enrichment analysis. In addition, a gene co‐expression network is constructed using a correlation metric. (c) The gene co‐expression network is decomposed spectrally to produce Fourier modes (FMs), which allow spot expression profiles to be represented in the frequency domain through graph Fourier transform (GFT). Low‐pass filtering is applied to reduce noise, and the frequency‐domain representations are then input into an autoencoder for further feature extraction. (d) During model training, the cell‐type compositions of pseudo‐spots are known, enabling the model to learn the relationships between gene expression and cell‐type compositions. After training, the model predicts the cell‐type compositions of real‐spots. (e) The predicted cell‐type composition is then used to regenerate higher‐quality pseudo‐spots, which serve as improved training data for the next iteration. (f) STAID enables spatial analysis by visualizing cell‐type distributions, quantifying cell‐type compositions in functional regions, assessing cell‐type colocalization, and identifying genes spatially associated with specific cell types for enrichment analysis.

STAID is designed to accurately map cell‐type distributions by integrating scRNA‐seq reference data with spatial transcriptomics data. The STAID workflow is illustrated in Figure 1. STAID takes spatial transcriptomics data as input along with a matched scRNA‐seq reference that includes cell‐type annotation information (Figure 1a). STAID first generates pseudo‐spots by repeatedly sampling multiple cells from the scRNA‐seq data, for which both gene expression profiles and cell‐type compositions are known. By learning the relationship between gene expression and cell‐type proportions from these pseudo‐spots, the trained model can then be applied to real ST data for cell‐type composition inference. Existing methods often generate pseudo‐spots entirely at random, producing unrealistic training data and reducing deconvolution accuracy. In contrast, STAID focuses on enhancing the quality of pseudo‐spot training data. Before generating pseudo‐spots, an enrichment analysis is performed to estimate the potential cell types present in each spot (Figure 1b, See “Methods”). Based on this cell‐type enrichment information, STAID generates pseudo‐spots by sampling cells only from the cell types likely present in each spot, thereby producing more realistic approximations of real‐spots and providing a more representative training set.

Next, a gene network is constructed based on co‐expression information in scRNA‐seq data to capture gene–gene relationships (Figure 1b). In this framework, the expression profiles of pseudo‐spots and real‐spots are represented as graph signals on the gene co‐expression network. The graph Fourier transform (GFT) is then applied to project these signals from the vertex domain to the frequency domain, followed by low‐pass filtering to denoise the data and retain biologically meaningful signals (See “Methods”). Low‐pass filtering in graph signal processing is widely adopted to enhance more valuable features, where low‐frequency components capture more stable global patterns, whereas high‐frequency components are more likely to represent noise [50, 51, 52, 53]. Unlike traditional approaches that treat genes as independent entities, GFT captures the underlying topological relationships among them. The resulting frequency‐domain representations (gene features transformed into Fourier modes, FMs) are then input into an autoencoder for further feature extraction (Figure 1c). The encoder outputs of pseudo‐spots are further passed through a multilayer perceptron (*MLP*) with a *Softmax* function as the last layer to predict cell‐type proportions (Figure 1d). Since the true cell‐type compositions of pseudo‐spots are known, the model is trained by minimizing the discrepancy between predicted and actual proportions. After training, the model is applied to real‐spots to infer their cell‐type compositions (Figure 1e).

Innovatively, STAID employs an iterative pseudo‐spot refinement strategy (Figure 1d,e, See “Methods”). In each iteration, the predicted cell‐type compositions of real‐spots guide the generation of new pseudo‐spots: for each real‐spot, the number of cells per cell type is determined according to the predicted proportions, ensuring that the pseudo‐spots more closely resemble the true composition of spatial transcriptomics data. Moreover, this iterative refinement progressively constrains the pseudo‐spot space toward biologically plausible compositions, improving both learning efficiency and predictive reliability. Higher‐quality pseudo‐spots are generated for subsequent model training, which in turn further enhances the quality of generated pseudo‐spots in the next iteration, creating a positive feedback loop, allowing the model to iteratively enhance its predictive power. More details are provided in the Methods section.

In summary, STAID offers a robust framework for cell‐type deconvolution in spatial transcriptomics based on scRNA‐seq reference data, integrating graph signal processing, and iterative pseudo‐spot refinement. The iterative pseudo‐spot generation strategy further enhances training data realism and model performance. Together, these innovations allow STAID to provide accurate and reliable cell‐type composition estimates across diverse spatial transcriptomics datasets, facilitating a deeper understanding of tissue organization and cellular heterogeneity (Figure 1f).

### 2.2 Comprehensive Benchmarking Illustrates the Superior Performance of STAID Over the Existing Methods

Since ground‐truth cell‐type proportions are not accessible, it is impractical to directly evaluate deconvolution performance in practice. In performance evaluation, a common approach is to generate synthetic spots by randomly sampling and combining multiple cells from scRNA‐seq data. While these synthetic spots share the same sequencing modality, the random aggregation of cells does not recapitulate the structured spatial organization and co‐localization patterns observed in real tissues. In this study, inspired by CytoSPACE [54], we leveraged a mouse brain Slide‐seqV2 dataset [55], which provides near single‐cell resolution. Each Slide‐seqV2 spot was substituted with a real single cell from the matched scRNA‐seq dataset to generate simulated sequencing‐based spatial transcriptomics data at single‐cell resolution. The tissue was then partitioned into spatial grids, and the cells within each grid were merged to construct synthetic spots, with their cell type compositions serving as ground truth.

**FIGURE 2:**
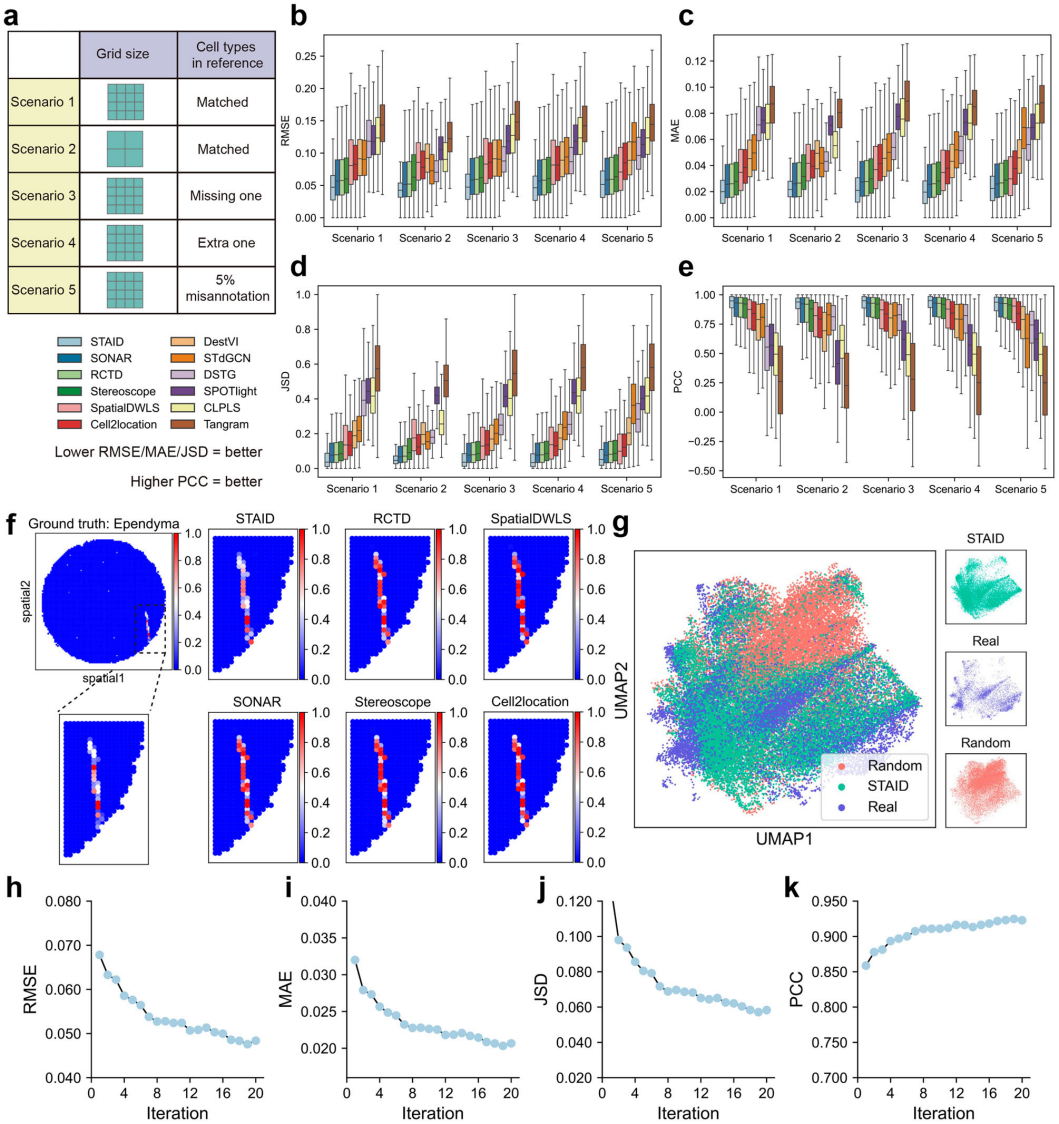
STAID outperforms existing methods in simulated scenarios and improves through iterative refinement. (a) Five scenarios were designed: Scenarios 1 and 2 used different grid sizes to represent varying spatial resolutions; Scenario 3 excluded a rare cell type present in spatial data from the scRNA‐seq reference; Scenario 4 included an extra cell type present in scRNA‐seq but absent in spatial data. Approximately 5% of cells in the reference dataset were randomly assigned incorrect cell type labels in Scenario 5. (b–e) Performance comparison at spot level across four evaluation metrics: *RMSE* (b), *MAE* (c), *JSD* (d), and *PCC* (e). (f) Comparison of predicted spatial distributions of Ependyma across methods. STAID most accurately recapitulated the ground‐truth linear distribution. (g) UMAP visualization of spots from three sources: STAID‐generated pseudo‐spots, randomly generated pseudo‐spots, and real‐spots. Pseudo‐spots produced by STAID showed greater consistency with the real‐spot distribution. (h,k) Evaluation metrics (*RMSE*, *MAE*, *JSD*, and *PCC*) at spot level across iterations. The prediction performance progressively improves as the number of iterations increases.

Next, we designed five scenarios to comprehensively evaluate the performance of our method (Figure 2a, See “Methods”). Scenarios 1 and 2 differ in grid size, reflecting different resolutions of spatial transcriptomics technologies, with Scenario 2 having a larger grid size. In Scenario 3, a rare cell type in the synthetic spatial transcriptomics data was removed from the reference data. In Scenario 4, we added an additional cell type that is present in the scRNA‐seq reference data but absent in the synthetic spatial transcriptomics data. Scenarios 3 and 4 were primarily used to assess how mismatches between the cell types in scRNA‐seq and spatial transcriptomics data affect method performance. In Scenario 5, to evaluate the tolerance of methods to cell type misannotation in the reference data, we introduced a simulation setting where approximately 5% of cells in the reference dataset were randomly assigned incorrect cell type labels. Eleven competitive deconvolution methods, SONAR [56], RCTD [40], Cell2location [39], SpatialDWLS [38], Stereoscope [57], DestVI [58], STdGCN [42], DSTD [41], SPOTlight [59], CLPLS [60] and Tangram [61], were selected for performance comparison with STAID. These methods cover the main methodological approaches in the field. Among these, some methods are statistical‐based, such as SONAR and RCTD; some are deep learning‐based, such as STdGCN and DSTD; and others are optimization‐based, such as SpatialDWLS and Tangram. For each method, simulated spatial transcriptomics data and the corresponding scRNA‐seq reference data were input, and the cell type composition of each spot was estimated. In the following, four evaluation metrics, *RMSE*, *MAE*, *JSD* and *PCC* were used to compare the differences or the similarity between predicted cell type compositions in each spot with ground truth at spot level (See “Methods”).

Across all scenarios, STAID consistently demonstrated the best performance and highest robustness, followed by SONAR, RCTD, and Stereoscope. In contrast, methods such as SPOTlight, CLPLS, and Tangram performed the worst (Figure 2b–e). From another perspective, we also characterized the spatial distribution patterns for each cell type. Accordingly, we compared the spatial distribution vector of each cell type with the ground truth to evaluate their similarity or discrepancy at the cell‐type level. In line with the previous comparison results, STAID consistently outperformed other methods, achieving the best performance across all cell type–specific spatial distribution analyses (Figure S1a–d), with statistically significant improvements in nearly all comparisons (Table S1). Take Ependyma as an example, a type of neuronal support cell that forms the epithelial lining of the brain ventricles and exhibits a line‐like distribution pattern in our datasets. STAID accurately inferred this linear distribution, with predictions most closely matching the ground truth. Other methods, except DSTG and CLPLS, also captured a line‐like pattern, but their results deviated to varying degrees from the true distribution (Figure 2f; Figure S1e). Another example, Astrocyte_Gja1, STAID likewise produced predictions consistent with the ground truth (Figure S2a).

These superior results can be attributed to STAID's iterative training strategy. In this design, pseudo‐spots are synthesized based on predictions from the previous iteration, yielding distributions that more closely resemble those of real spots. The improved quality of such training data in turn benefits model learning and prediction. To validate this mechanism, we compared pseudo‐spots synthesized from prior predictions with those generated by random cell aggregation (Figure 2g) and found that the former were more consistent with the real distribution. Furthermore, by examining outputs across successive iterations against the ground truth, we observed clear improvements in prediction performance (Figure 2h–k; Figure S2b–e), demonstrating that our iterative strategy is both effective and well‐founded. To quantify the contributions of key components in STAID, we conducted a comprehensive ablation study by systematically removing each module, including pseudo‐spot refinement, graph Fourier transform, the autoencoder module, and random spot supplementation (Figure S3a–d). The results show that these components contribute to performance improvement, with the full model consistently achieving the best results, demonstrating the effectiveness of the STAID framework.

In addition, we further evaluated STAID on two single‐cell‐resolution spatial transcriptomics platforms, MERFISH and SeekSpace (Figures S4a and S5a). To provide a more rigorous assessment, the datasets used as reference data were derived from tissue sections that were independent of those used for simulated spot generation. Across all evaluation metrics (*RMSE*, *MAE*, *JSD*, and *PCC*), STAID consistently achieved the best performance compared with competing methods, with statistically significant improvements in nearly all comparisons (Figures S4b–i and S5b–i and Tables S2 and S3). Furthermore, visualization of representative cell types demonstrated that the spatial distributions inferred by STAID showed improved concordance with ground‐truth patterns, exhibiting more biologically plausible spatial organization (Figures S6a–c and S7a–c). Overall, these results demonstrate the strong robustness and high accuracy of STAID across diverse spatial transcriptomics platforms.

In the following, sensitivity analysis of key parameters further supports the robustness of our method, indicating stable performance across different parameter settings (Figure S8a–d). To evaluate robustness to initial cell‐type estimates, we conducted simulation experiments in which a subset of cell‐type labels enriched in the initial estimates were randomly flipped and the results demonstrate that STAID remains robust (Figure S9a). We also evaluated the computational efficiency and scalability of STAID on spatial transcriptomics datasets with different scales (Figure S10a,b). The results also show that STAID maintains stable runtime and memory usage across datasets of increasing size compared with the existing methods.

**FIGURE 3:**
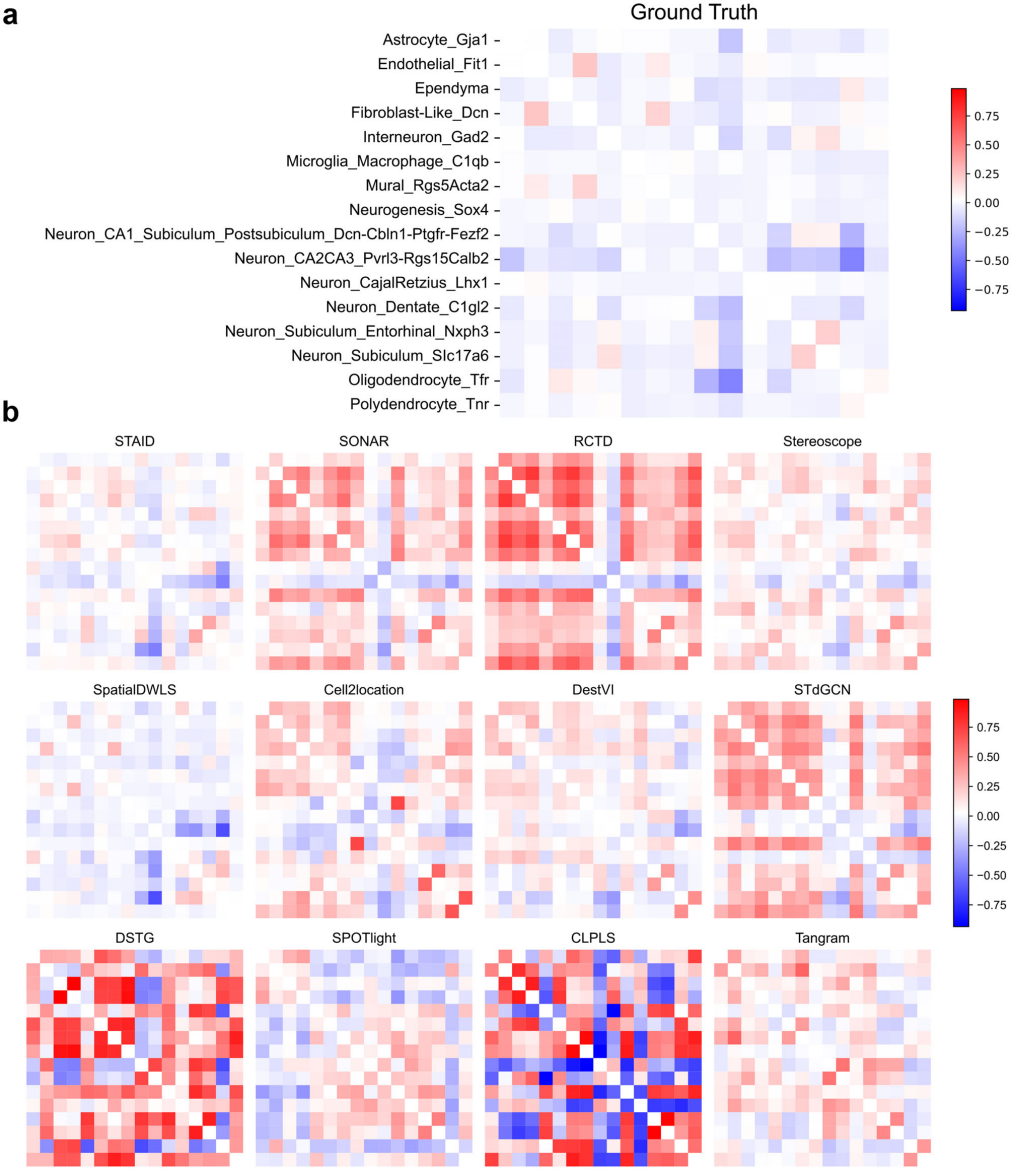
Evaluation of cell–cell co‐localization patterns. (a) Ground‐truth pairwise spatial correlations between cell types in Scenario 2. The row and column orders of the heatmap are aligned to be consistent. (b) Comparison of predicted pairwise correlations across methods. STAID and SpatialDWLS best recapitulated the ground truth, while SONAR, RCTD, and STdGCN produced excessive false positives, and DSTD and CLPLS introduced both false positives and false negatives. The row and column names of these heatmaps were consistent with ground truth.

Finally, we evaluated the biological relevance of cell‐type predictions by examining cell‐type co‐localization, a critical aspect of many spatial transcriptomics analyses. Accurate characterization of co‐localization can uncover functional interactions between neighboring cell types [62, 63, 64], such as immune infiltration, tumor–stromal crosstalk, or neuron–glia communication. Co‐localization relationships are typically assessed by computing spatial correlations between pairs of cell types. In Scenario 2, where the spot size was relatively larger and more suitable for co‐localization analysis, we calculated pairwise spatial correlations using Spearman's coefficient on the synthetic spatial data. The results show that STAID and SpatialDWLS produced outcomes most consistent with the ground truth, thereby better preserving authentic patterns of cell–cell spatial association (Figure 3a,b; Figure S11a), although SpatialDWLS did not perform notably well in previous evaluations. In some cell‐type pairs, such as Neuron_Subiculum_Slc17a6 and Interneuron_Gad2, STAID and SpatialDWLS showed comparable correlation coefficients with the ground truth; however, SpatialDWLS exhibited larger spatial pattern differences (Figure S12a–e). In contrast, SONAR, RCTD, and STdGCN tended to generate excessive false‐positive correlations, likely reflecting their limited capacity to accurately resolve cellular compositions within spots. DSTD and CLPLS introduced both false‐positive and false‐negative associations. These findings indicate that STAID more faithfully captures cell–cell co‐localization patterns, highlighting its ability to preserve biologically meaningful spatial relationships compared with other methods.

Overall, these findings demonstrate that STAID not only improves deconvolution accuracy but also enables deeper insights into spatial cellular organization and co‐localization.

### 2.3 STAID Accurately Reveals Tumor Populations and the Spatial Organization of Tumor Microenvironment in Breast Cancer

**FIGURE 4:**
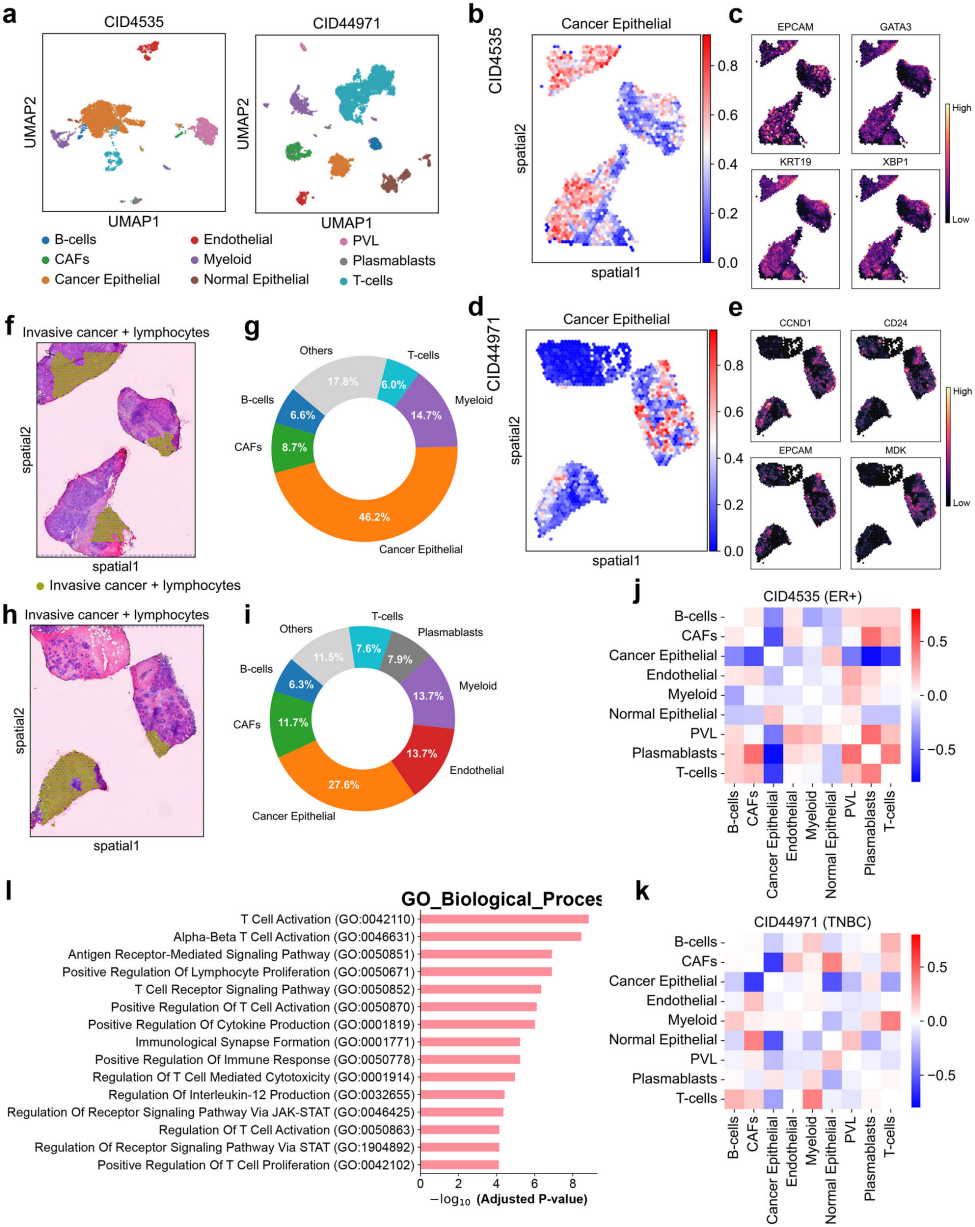
STAID accurately reveals tumor cells and the spatial organization of the tumor microenvironment in breast cancer. (a) UMAP visualization of the scRNA‐seq reference dataset with cell‐type annotations. (b) Predicted tumor epithelial cell distribution in CID4535. (c) Spatial distribution of tumor epithelial–specific DEGs in CID4535. (d) Predicted tumor epithelial cell distribution in CID44971. (e) Spatial distribution of tumor epithelial–specific DEGs in CID44971. (f) Annotated “invasive cancer + lymphocytes” region in CID4535. (g) Cell‐type composition within the annotated region in CID4535. (h) Annotated “invasive cancer + lymphocytes” region in CID44971. (i) Cell‐type composition within the annotated region in CID44971. (j) Heatmap of cell‐type co‐localization analysis based on STAID predictions in CID4535. (k) Heatmap of cell‐type co‐localization analysis based on STAID predictions in CID44971. (l) Gene Ontology enrichment analysis of genes spatially associated with T cells in CID44971.

To further demonstrate the utility of STAID in cancer research with spatial transcriptomics, we applied it to two breast cancer Visium samples [65], CID4535 (estrogen receptor–positive, ER+) and CID44971 (triple‐negative breast cancer, TNBC). This case allowed us to assess how well STAID can resolve cellular compositions while capturing the spatial organization of heterogeneous tumor microenvironments. Due to the intrinsic heterogeneity of tumor tissues, we utilized the matched single‐cell reference datasets from the same patients in the original study, along with their corresponding cell‐type annotations (Figure 4a). We first focused on evaluating STAID's ability to accurately identify tumor epithelial cells. Because tumor cells exhibit heterogeneity across samples, their marker genes can vary. To address this, we performed differential expression (DEG) analysis on the matched scRNA‐seq reference dataset to identify tumor epithelial cell–specific genes (Figure S13a,b). We then compared the spatial distribution of tumor cells predicted by STAID with the expression patterns of these DEGs to assess their concordance. Notably, this agreement was evident in both CID4535 and CID44971 (Figure 4b–e), demonstrating that STAID reliably recovers the spatial localization of tumor epithelial cells while faithfully capturing their molecular identity and heterogeneity within the tumor microenvironment. In addition, STAID can also distinguish tumor epithelial cells from normal epithelial cells. The two populations exhibit distinct spatial distributions, with normal epithelial cells enriched in one region and tumor epithelial cells in others (Figure S14a), and these spatial distribution patterns are supported by DEGs (Figure S14b–d).

In the original study, tissue slices were annotated based on histopathology, including regions such as “invasive cancer”, “stroma”, and “invasive cancer + lymphocytes” (Figure S13c,d). Our study concentrated on the “invasive cancer + lymphocytes” regions, which contain both tumor cells and infiltrating lymphocytes, offering an opportunity to investigate spatial organization and interactions within the tumor–immune microenvironment, as observed in tumor heterogeneity studies [66, 67, 68]. We quantified the cell‐type composition in these regions for both samples. In CID4535, the region was predominantly composed of tumor epithelial cells, myeloid cells, CAFs, B cells, and T cells (Figure 4f,g; Figure S15a), whereas CID44971 additionally included endothelial cells and plasmablasts (Figure 4h,i; Figure S16a). These differences underscore the inherent heterogeneity of the tumor microenvironment across patients.

We next performed spatial co‐localization analysis using STAID deconvolution results by calculating Spearman rank correlation coefficients between each pair of cell types to infer spatial co‐localization patterns (Figure 4j,k). In both samples, tumor epithelial cells exhibited strong negative correlations with several other cell types, reflecting their spatial segregation within the tissue. Notably, the negative correlation between tumor epithelial cells and T cells was weaker in CID44971 (TNBC) compared to CID4535 (ER+), consistent with previous reports that triple‐negative breast cancers are characterized by higher levels of tumor‐infiltrating lymphocytes (TILs) and a more immune‐infiltrated tumor microenvironment [69, 70, 71]. To further investigate the functional programs of T cells in CID44971, we computed spatial correlations between T cells and all other genes to identify genes with strong spatial co‐localization (See “Methods”). Gene Ontology (GO) enrichment analysis of these genes, as expected, revealed significant enrichment for processes related to T cell activation, T cell receptor signaling, lymphocyte proliferation, positive regulation of T cell activation, and cytokine production (Figure 4l). These findings indicate that genes spatially associated with T cells are functionally linked to core immune activities, highlighting active immune responses within the TNBC tumor microenvironment.

In summary, STAID not only reconstructed spatial cellular compositions with high accuracy but also provides insights into tumor heterogeneity and the immune microenvironment, underscoring its value for cancer spatial transcriptomics studies.

### 2.4 STAID Efficiently Deciphers Spatial and Hierarchical Organization of Cell Types in Human Embryonic Limb Development

**FIGURE 5:**
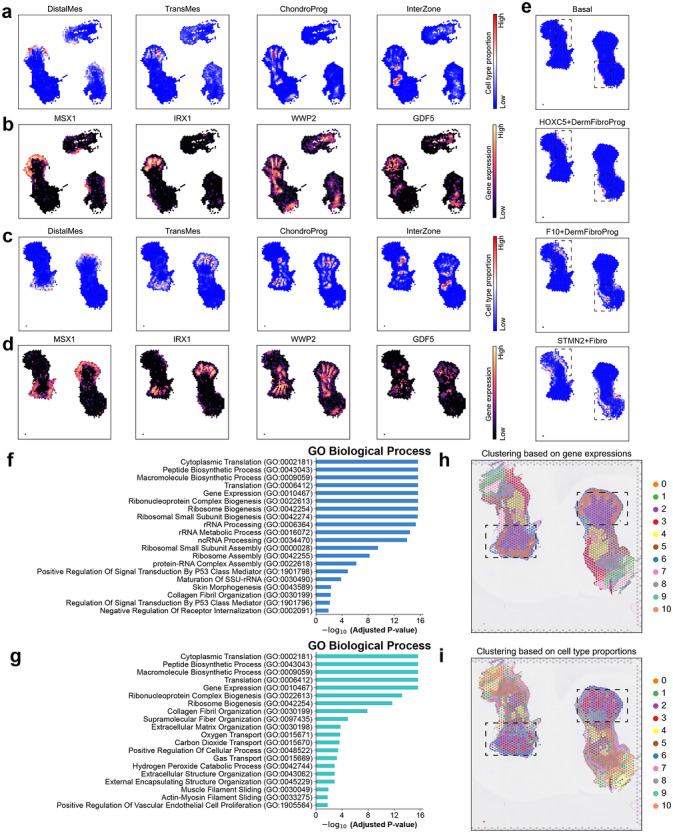
STAID reconstructs spatial distributions of progenitor populations in human embryonic limb buds. (a) Spatial distributions of four cell types at 5.6 PCW. (b) Corresponding marker gene expression patterns for the cell types shown in (a). (c) Spatial distributions of four cell types at 6.2 PCW (d) Corresponding marker gene expression patterns for the cell types shown in (c). (e) Spatial distributions of four fibroblast‐related cell types in the limb bud, showing a clear layered arrangement from basal cells at the outermost edge, followed by HOXC5+DermFibroProg, F10+DermFibroProg, and STMN2+Fibro. (f) Gene Ontology enrichment analysis of genes spatially correlated with HOXC5+DermFibroProg, highlighting enrichment in translation, ribosome biogenesis, and general gene expression processes. (g) GO enrichment analysis of genes spatially correlated with F10+DermFibroProg, showing enrichment in biosynthetic processes, extracellular matrix organization, and vascular‐related functions, including positive regulation of vascular endothelial cell proliferation and gas transport. (h) Leiden clustering of spots at PCW6.2 based on gene expression profiles. Clustering based on gene expression alone does not fully resolve key tissue structures. (i) Leiden clustering of spots at PCW6.2 using STAID‐derived cell type proportions as clustering features. Cell‐type–based clustering accurately delineates tissue structures, including the autopod region, digital (cluster 2), interdigital (cluster 5), and distal mesenchyme (cluster 0).

Human limb buds emerge by the end of the fourth post‐conception week (PCW) and progressively develop into arms and legs during the first trimester. Limb development is a highly dynamic and complex process in which a relatively homogeneous limb bud differentiates into multiple cell types to form the various limb tissues [72]. Accurate deconvolution of spatial transcriptomics data is therefore essential for mapping cell‐type distributions and understanding this developmental process. In the following, we applied STAID to two human limb Visium datasets at 5.6 and 6.2 PCW, using a matched scRNA‐seq reference data [73] with cell type annotation information (Figure S17a). At these early stages, most enriched populations were progenitors [73]. STAID accurately reconstructed major embryonic limb cell types, including Distal Mesenchyme (DistalMes), Transitional Mesenchyme (TransMes), Chondrogenic Progenitors (ChondroProg), and InterZone, with spatial patterns closely matching the expression of marker genes selected in the original study (Figure 5a–d). For instance, ChondroProg cells, marked by *WWP2*, were enriched in distal regions corresponding to future phalanges, whereas DistalMes cells, marked by *MSX1*, localized to the distal tip of the limb bud, consistent with future digit formation and interdigital regions [73, 74]. In addition, these spatial distribution patterns are also consistent with in situ spatial transcriptomics (ISS) data [73, 75, 76] (Figure S18a). These reconstructed spatial patterns of progenitor populations confirm the accuracy of STAID in mapping early human limb development.

We also observed an ordered spatial arrangement among several cell types in the embryonic limb (Figure 5e; Figure S19a). Basal cells were predominantly located at the outermost edge, followed inward by HOXC5+ Dermal Fibroblast Progenitors (HOXC5+DermFibroProg) and then F10+ Dermal Fibroblast Progenitors (F10+DermFibroProg). Notably, F10+DermFibroProg also exhibited spatial co‐localization with STMN2+ Fibroblasts (STMN2+Fibro), suggesting that these fibroblast populations may cooperate to form more complex structures and functions. These spatial patterns were also observed at 5.6 PCW and ISS data (Figures S18b, S19b, and S20a), suggesting the potential coordinated roles of fibroblast populations in tissue formation during early limb development. By leveraging STAID, we were able to resolve these ordered spatial arrangements, capturing subtle differences between closely related progenitor populations.

To further investigate the differences between the two DermFibroProg populations, we analyzed genes spatially correlated with HOXC5+DermFibroProg and F10+DermFibroProg. Genes associated with HOXC5+DermFibroProg were mainly enriched in translation, ribosome biogenesis, and general gene expression processes, reflecting active protein synthesis (Figure 5f,g). In contrast, F10+DermFibroProg‐associated genes were enriched not only in similar biosynthetic processes but also in extracellular matrix organization and vascular‐related functions, including positive regulation of vascular endothelial cell proliferation, gas transport, and oxygen and carbon dioxide transport. While both fibroblast progenitors are biosynthetically active, F10+DermFibroProg potentially plays additional roles in tissue structure, vascular development, and local metabolic processes.

Finally, we evaluated whether spot‐level cell type compositions could serve as features for spot clustering, which is a critical step for ST data analysis, to delineate tissue structures at 6.2 PCW. Using the widely adopted Leiden algorithm [77] directly, we performed spot clustering based on gene expression information and cell‐type composition information respectively. Remarkably, even with this straightforward approach, clustering based on cell‐type composition information effectively recapitulated tissue architecture (Figure 5h,i). For example, in the autopod region, digital (cluster 2), interdigital (cluster 5), and distal mesenchyme (cluster 0) were clearly distinguished in spot clustering results based on cell‐type proportion information inferred by STAID (Figure 5i), whereas clustering based on gene expression failed to resolve these structures. Similar patterns were observed in the 5.6 PCW sample (Figure S20b,c). Such segmentation was supported by the effective prediction of cell‐type proportions by STAID (Figure S21a–l). These results demonstrate that STAID‐derived spot‐level cell type composition information also provides robust features for clustering in some cases, enabling precise delineation of spatial structures. Given that most current spatial clustering approaches rely solely on gene expression, our findings suggest that incorporating cell type composition may substantially enhance tissue architecture characterization.

In summary, STAID accurately reconstructed the spatial organization of key progenitor populations in the human embryonic limb, capturing both their biologically meaningful distributions and ordered spatial arrangements.

### 2.5 STAID Resolves Intestinal Spatial Cellular Organization and Characterizes the Complex Cell‐Type Composition of TLS‐Like Immune Niches in Human Crohn's Disease

**FIGURE 6:**
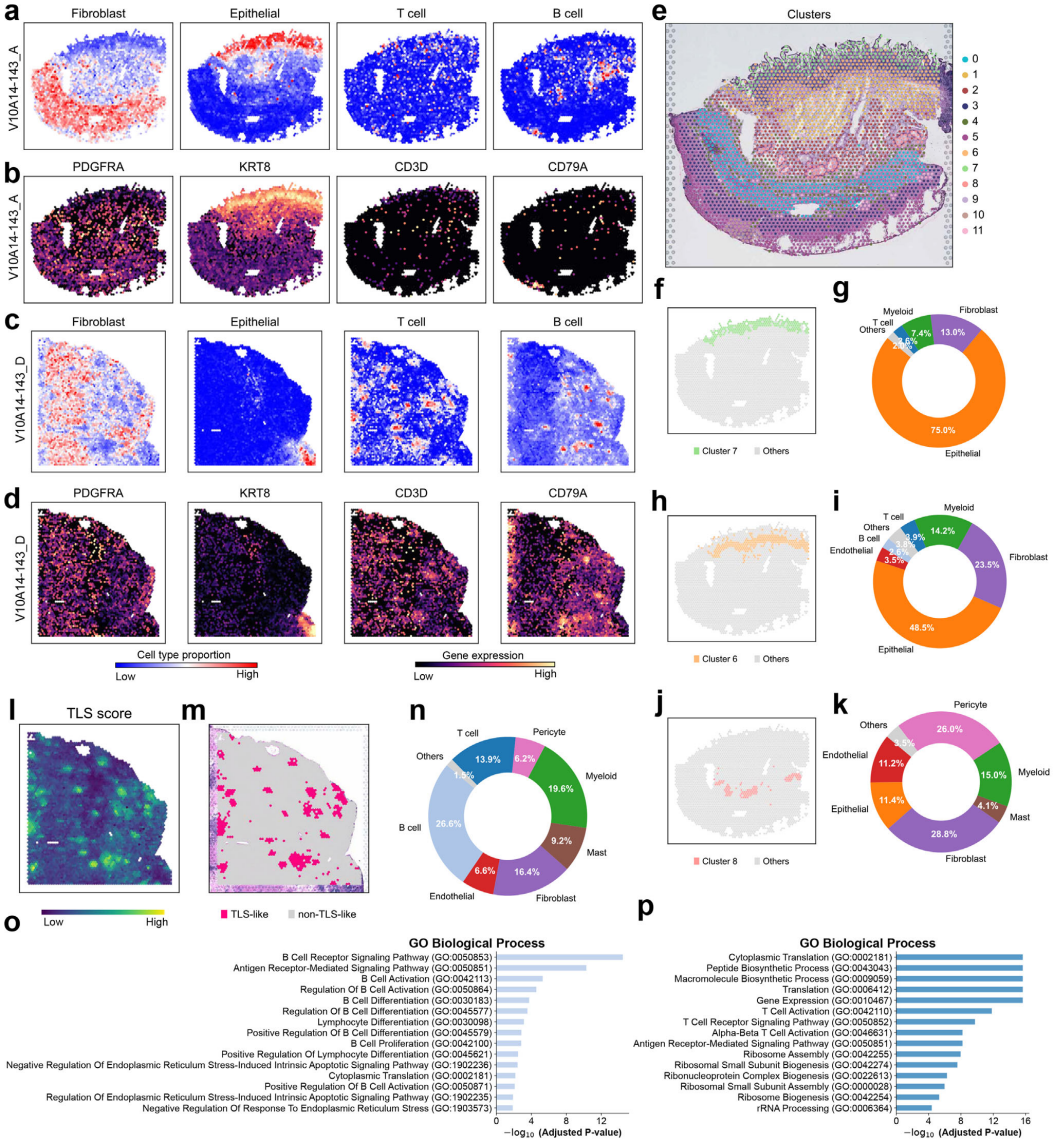
STAID resolves intestinal spatial cellular organization and characterizes cell‐type distribution patterns underlying TLS‐like immune niches in Crohn's disease. (a) Spatial distributions of fibroblasts, epithelial cells, T cells, and B cells in the adjacent non‐stricture sample V10A14‐143_A predicted by STAID. (b) Spatial expression patterns of canonical lineage marker genes in V10A14‐143_A: *PDGFRA* (fibroblasts), *KRT8* (epithelial cells), *CD3D* (T cells), and *CD79A* (B cells). (c) Spatial distributions of fibroblasts, epithelial cells, T cells, and B cells in sample V10A14‐143_D predicted by STAID. (d) Spatial expression patterns of *PDGFRA*, *KRT8*, *CD3D*, and *CD79A* in V10A14‐143_D. (e) Leiden clustering of spatial spots in V10A14‐143_A (clusters 0–11). (f,h,j) Spatial patterns of clusters 7, 6, and 8, respectively. (g,i,k) Cell‐type compositions of clusters 7, 6, and 8, respectively. (l) Spatial map of TLS scores in V10A14‐143_D, defined as the mean log‐expression of TLS signature genes. (m) Spatial annotation of TLS‐like and non‐TLS‐like niches in V10A14‐143_D based on TLS score thresholding. (n) Quantified cell‐type proportions within TLS regions in V10A14‐143_D derived from STAID predictions. (o,p) Gene Ontology enrichment analysis of B cells (o) and T cells (p) in V10A14‐143_D.

Crohn's disease (CD) is a chronic inflammatory bowel disease characterized by recurrent intestinal inflammation, progressive extracellular matrix deposition, and irreversible stricture formation [78]. In this study, we applied STAID to characterize the spatial cellular composition of two stricture sections (V10A14‐143_C, V10A14‐143_D) and one adjacent non‐stricture section (V10A14‐143_A) from a CD patient [19] (Figures S22a,b and S23a) on Visium platform. To validate the accuracy of our deconvolution results, we compared the predicted spatial proportions of major cell types with the expression patterns of their canonical marker genes (Figure 6a–d; Figure S24a–d). Specifically, the predicted epithelial regions aligned with *KRT8* expression, while fibroblast populations co‐localized with the fibroblast marker *PDGFRA*. Similarly, the predicted spatial distributions of T cells and B cells faithfully mirrored the expression territories of *CD3D* and *CD79A*, respectively.

Building upon the observation that the non‐stricture mucosa section exhibits organized architecture in the Hematoxylin and Eosin (H&E) image (Figure S24e), we performed clustering analysis (Figure 6e) on this tissue section based on gene expression profiles and analyzed cell‐type abundances within clusters. At the luminal surface, the mucosal compartment exhibits a stratified organization consisting of Cluster 7 and Cluster 6. The outermost layer (Cluster 7) is specifically localized to the apical villus tips and is enriched for epithelial cells (∼75%), a key component of the mucosal barrier involved in nutrient absorption and frontline host defense [79] (Figure 6f,g). Immediately basolateral to this barrier lies Cluster 6, which occupies the subepithelial lamina propria [80]. Within this niche, epithelial content decreases as myeloid cells and tissue fibroblasts expand, forming an important immune‐surveillance interface where luminal antigens are sampled and mucosal tolerance is maintained [81] (Figure 6h,i). We observed prominent vascular structures in the submucosal layer, which corresponds to Cluster 8. STAID revealed that this cluster is primarily composed of endothelial cells, pericytes, and fibroblasts (Figure 6j,k). This cellular composition is consistent with previous findings [82, 83].

In the two stricture samples, STAID revealed a massive accumulation of B cells and T cells, suggesting the presence of TLS‐like immune niches, which have been reported in chronically inflamed tissues and may contribute to sustained local immune responses in Crohn's disease [84]. We calculated a TLS score based on the mean log‐expression of a validated signature gene set [85] (Table S4) in V10A14‐143_D, and TLS‐like regions were subsequently delineated based on this score (Figure 6l,m; See “**Methods**”). Within these regions, both B cells and T cells are highly enriched (Figure 6n), consistent with the canonical cellular composition of lymphoid tissues [86]. Notably, the presence of endothelial cells suggests the formation of high endothelial venule (HEV)‐like vasculature, which is known to support continuous lymphocyte recruitment [87]. These findings were independently replicated in V10A14‐143_C, confirming the spatial stability and deconvolution reproducibility of STAID (Figure S24f–h). Furthermore, functional enrichment analyses of genes whose spatial expression patterns highly correlate with B cell and T cell distributions (Figure 6o,p) align well with the cellular functions. These results demonstrate that STAID accurately captures the localized biological activities of adaptive immune cells within complex diseased microenvironments.

In summary, by accurately resolving localized cell‐type distribution patterns, our method provides a robust framework for deconstructing the spatial cellular architecture and heterogeneity underlying Crohn's disease.

## 3 Discussion

In this study, we present STAID, a unified deep learning framework that couples iterative pseudo‐spot refinement with neural network training through a feedback loop and exploits gene co‐expression information by GSP to model higher‐order interactions, achieving accurate and robust cell‐type deconvolution in spatial transcriptomics. By persistently generating pseudo‐spots that closely resemble real data and establishing a virtuous cycle, STAID provides a high‐quality training set that accurately reflects the characteristics of the target tissue compositions. The usage of GSP on gene co‐expression information efficiently captures biologically meaningful gene–gene relationships while reducing noise. These strategies facilitate the deep learning model to effectively learn complex mappings between gene expression and cell‐type proportions. Comprehensive benchmarking on simulated datasets demonstrates that STAID consistently outperforms existing deconvolution methods, achieving superior accuracy in predicting cell‐type distributions and cell‐type co‐localization relationships.

Applications in human breast cancer, embryonic limb, and human Crohn's disease datasets further underscore STAID's utility in spatial transcriptomics studies. In breast cancer tissues, STAID accurately reconstructed the spatial distribution of tumor cells, revealing biologically meaningful co‐localization patterns such as T cell infiltration and spatial segregation of tumor cells. In human embryonic limb datasets, STAID effectively captured the ordered spatial distributions of key progenitor populations, reflecting the hierarchical organization of developing tissues, and demonstrated that cell‐type composition information can more accurately delineate tissue structures compared with clustering based solely on gene expressions. In human Crohn's disease datasets, STAID profiled spatial cell‐type distribution patterns across intestinal tissues, capturing the cellular organization of epithelial, mucosal, and vascular compartments. These findings illustrate STAID's ability to provide interpretable insights into both tissue architecture and cellular heterogeneity across diverse biological contexts, facilitating a deeper understanding of complex spatial organizations.

The effectiveness of STAID stems from two key innovations. First, the comprehensive integration of iterative pseudo‐spot refinement within deep learning to ensure biologically realistic training samples, and frequency‐domain encoding of gene‐wise relationships to enhance the reliability and generalizability of cell‐type composition inference. Second, by generating pseudo‐spots guided by prior predictions, the training data progressively aligns with the true spatial organization of tissues, thereby enhancing model robustness and predictive accuracy. In our previous study [88], we conducted graph Fourier transform on spot adjacency graph to represent genes. Here, STAID applies gene co‐expression networks to represent spots, where GSP techniques effectively facilitate the extraction of biologically meaningful signals.

Despite these advantages, there remain opportunities to further improve the performance. First, STAID predicts cell‐type proportions at the spot level, which does not reach single‐cell resolution. This limitation could be addressed by integrating STAID with single‐cell resolution inference methods such as HistoCell [89] and CytoSPACE [54]. Second, incorporating complementary modalities, including histological or multi‐omics data, may further improve the identification of cell‐type compositions. Third, the global co‐expression construction strategy may filter out partially co‐expressed information in some subpopulations [90]. Future work could explore cell‐type‐aware or stratified co‐expression modeling to address this issue. Lastly, more advanced neural network architectures, such as attention‐based and diffusion models, could be employed in the future to further improve the prediction of cell‐type proportions.

Overall, STAID provides a versatile framework for delineating spatial cellular compositions, accurately mapping cell‐type distributions, and offering valuable insights into tissue organization and cellular interactions across diverse biological contexts.

## 4 Methods

### 4.1 Data Preprocessing and Feature Selection

To ensure consistency for downstream modeling, raw count matrix was employed for processing scRNA‐seq data. The initial processing involved first normalizing the raw counts by total count using the Scanpy [91] with *scanpy.pp.normalize_total* function and subsequently performing log‐transformation using *scanpy.pp.log1p*. Following this, feature selection was performed exclusively on the scRNA‐seq data: Differentially Expressed Genes (DEGs) across the annotated cell types were identified via the Wilcoxon rank‐sum test, implemented using the *scanpy.tl.rank_genes_groups* function (*method = ‘wilcoxon’*). These identified DEGs served solely to define the discriminative features required for cell type identification. In sharp contrast, all subsequent analyses, including cell type enrichment analysis and pseudo‐spot generation, commenced with the original raw count matrix of the scRNA‐seq data.

### 4.2 Spatial Cell Type Enrichment Analysis

To infer the potential cell types in each spatial spot, we applied a method inspired by the MIA algorithm [92]. Specifically, spatial spots were pre‐clustered first using the standard Scanpy workflow, including normalization, log‐transformation, PCA‐based dimensionality reduction, and Leiden clustering to define spatial domains. Differentially expressed genes (DEGs) for each spatial cluster were then identified using the same procedure as applied to the scRNA‐seq data. For each scRNA‐seq–defined cell type *k* and each spatial cluster, a hypergeometric test was performed to quantify the enrichment of cell type–specific DEGs within the cluster‐specific DEG set. Cell types with *p* < 0.05(default threshold) were considered significantly enriched in the corresponding spatial cluster. Finally, each spatial spot inherited the enriched cell‐type annotations from the cluster to which it belonged, providing biologically informed prior information for the subsequent generation of initial pseudo‐spots.

### 4.3 Pseudo‐Spot Generation Guided by Enrichment Priors

Most spatial transcriptomics techniques lack ground‐truth information on the true cell type composition at each spatial location, making reliable training labels unavailable. A common strategy is to synthesize pseudo‐spots from scRNA‐seq data by randomly selecting and aggregating individual cells. However, even when considering only which cell types co‐occur, the number of possible combinations increases exponentially, because each additional cell type doubles the space of possible co‐occurrence patterns. This accounts only for the discrete combinations. Within each combination, continuously varying cell‐type proportions further expands the diversity of possible pseudo‐spots, creating an immense compositional space. As a result, random sampling is unlikely to cover the biologically plausible regions of this space that are observed in real tissues. Therefore, a fully random pseudo‐spot generation strategy may be inherently suboptimal for model training.

Unlike other methods, STAID generated pseudo‐spots that closely resemble real spatial spots, providing high‐quality training data that improve model learning and ultimately enable more accurate and reliable estimation of cell type proportions. The generation strategy differed between the initial prediction and later iterative steps. During the initial prediction stage, STAID leveraged the results of prior cell‐type enrichment analysis: for each real spatial spot, the method identifies the set of potentially enriched cell types and constructs pseudo‐spots by sampling cells exclusively from these candidates. Specifically, during the initial pseudo‐spot construction stage, we leverage prior cell‐type enrichment analysis, which provides, for each real spatial spot, a set of candidate cell types that are likely to be present. Based on this information, a candidate cell pool is first constructed by extracting the corresponding cells that belong to these enriched cell types from the scRNA‐seq reference dataset. For each pseudo‐spot, the total number of cells is first randomly sampled from a uniform distribution within a predefined range (*min_cells* to *max_cells*, default: 1–8) from the cell pool. These sampled cells are then randomly selected from the candidate pool and aggregated to form a pseudo‐spot. To further increase diversity, a subset of pseudo‐spots (default 30%) is generated by sampling from all cell types without restriction. In this way, by default, a total of 5,000 pseudo‐spots were generated at this stage. This process yielded the gene expression matrices of pseudo‐spots and real spots, denoted as $F_{pseudo}=\left[f_{pseudo}^{1};f_{pseudo}^{2},\;\text{…},\;f_{pseudo}^{n_{1}}\right]\in R^{n_{1}\times m}$ and $F_{real}=\left[f_{real}^{1};f_{real}^{2},\;\text{…},\;f_{real}^{n_{2}}\right]\in R^{n_{2}\times m}$, where *n*_1_ is the number of pseudo‐spots, *n*_2_ is the number of real‐spots and *m* is the number of genes. Each $f_{s}^{i}$ represents the log‐normalized gene expression vector of spot *i*, with s∈{real,pseudo}.

During subsequent iterations, pseudo‐spot construction was adaptively guided by the cell‐type compositions inferred from all real spots in the previous iteration, rather than relying on a fixed or randomly generated sampling scheme. Specifically, only cell types identified in the preceding iteration were considered, and the number of sampled cells per type was proportional to their inferred fractions, thereby constraining pseudo‐spot generation toward biologically plausible compositions. To preserve biological variability and avoid over‐constraining the sampling process, a small stochastic perturbation was introduced to the inferred proportions, and the total number of cells per pseudo‐spot was uniformly sampled within predefined bounds. A subset of pseudo‐spots (default 30%) was generated by sampling from all cell types without restriction. In this way, STAID enables a progressive refinement of the pseudo‐spot distribution across iterations, effectively coupling pseudo‐spot construction with model learning into a unified framework.

### 4.4 Gene Co‐Expression Network Construction and Spectral Decomposition

A gene co‐expression network was constructed from scRNA‐seq data to capture higher‐order dependencies among genes. For each gene pair *i*, *j*, let $x^{i}=\left(x_{1}^{i},\;x_{2}^{i},\;\text{…},\;x_{n}^{i}\right)$ and $x^{j}=\left(x_{1}^{j},\;x_{2}^{j},\;\text{…},\;\;x_{n}^{j}\right)$ denote log‐normalized expression vectors across *n* cells, where *n* is the number of cells in scRNA‐seq, and calculate the similarity/correlation coefficient between gene *i* and gene *j* by

$$a_{ij}=corr\left(x^{i},x^{j}\right)$$

where the *corr*(·,   ·) denotes cosine similarity metric (default), Pearson correlation coefficient or Spearman rank correlation coefficient. Low‐weight edges ($a_{\mathrm{ij}}$< 0.3,  default) were removed to retain significant associations. The weighted adjacency matrix $A=\left(a_{ij}\right)\in R^{m\times m}$ and the corresponding degree matrix $D=diag(d_{1},d_{2},\;\text{…},\;d_{m})$​, where $d_{i}=\sum_{j}a_{ij}$ are used to compute the normalized graph Laplacian matrix:

$$L=I-D^{-1/2}AD^{-1/2}\in R^{m\times m}$$

where $I\in R^{m\times m}$ is an identity matrix.

Spectral decomposition of L by $L=U\Lambda U^{T}$ produced eigenvectors $U=(u_{1},u_{2},\text{…},u_{m})$ and eigenvalues $\Lambda =\mathrm{diag}(\lambda_{1},\;\lambda_{2},\;\cdots ,\lambda_{m})$ forming a graph Fourier basis. Eigenvectors corresponding to low eigenvalues represent low‐frequency Fourier modes, capturing smooth, global trends in gene expression, whereas high‐frequency modes represent oscillatory patterns. These spectral components provide systematic topological descriptors of the gene expression manifold, which, when integrated into STAID, enable the model to effectively capture complex, higher‐order relationships among genes beyond simple pairwise correlations.

### 4.5 Graph Fourier Transform, Low‐Pass Filtering, and Batch Correction

For each spot *k* and type s∈{pseudo,real}, let $f_{s}^{k}=(f_{s,\;1}^{k},\;f_{s,2}^{k},\;\cdots ,f_{s,m}^{k})$ be the gene expression vector. The graph Fourier transform is computed as:

$${\hat{f}}_{s}^{k}=({\hat{f}}_{s,\;1}^{k},\;{\hat{f}}_{s,\;2}^{k},\;\cdots ,{\hat{f}}_{s,\;m}^{k})=U^{T}f_{s}^{k}$$

A low‐pass filtering attenuates high‐frequency components to reduce technical noise [88]:

$${\bar{f}}_{s}^{k}=\left(\frac{1}{1+c\lambda_{1}}{\hat{f}}_{s,\;1}^{k},\;\frac{1}{1+c\lambda_{2}}{\hat{f}}_{s,\;2}^{k},\;\text{…},\frac{1}{1+c\lambda_{n}}{\hat{f}}_{s,\;m}^{k}\right)$$

followed by L2 normalization:

$${\tilde{f}}_{s}^{k}=\left({\tilde{f}}_{s,\;1}^{k},\;{\tilde{f}}_{s,\;2}^{k},\text{…},\;{\tilde{f}}_{s,\;m}^{k}\right)=\frac{{\bar{f}}_{s}^{k}}{∥{\bar{f}}_{s}^{k}{∥}_{2}}$$

where, *c*(default) serves as a smoothing parameter modulating the low‐pass filter intensity. Subsequently, batch effect correction was applied to the low‐pass filtered spectral coefficients using Scanorama [93] or ComBat [94], while other batch correction methods [95, 96, 97] can also be incorporated within this framework, depending on the degree of divergence between pseudo‐spots and real‐spots. The corrected spectral representations ${\tilde{F}}_{pseudo}=\left[{\tilde{f}}_{pseudo}^{1};{\tilde{f}}_{pseudo}^{2},\;\text{…},\;{\tilde{f}}_{pseudo}^{n_{1}}\right]\in R^{n_{1}\times m}$ and ${\tilde{F}}_{real}=\left[{\tilde{f}}_{real}^{1};{\tilde{f}}_{real}^{2},\;\text{…},\;{\tilde{f}}_{real}^{n_{2}}\right]\in R^{n_{2}\times m}$​ then served as input features for semi‐supervised deep learning‐based cell type deconvolution. Frequency‐domain features reduce noise, capture multi‐scale expression patterns, and yield compact, interpretable signals, ensuring consistency across datasets while preserving biologically meaningful information [98].

### 4.6 Feature Autoencoder

To obtain representative embeddings of pseudo‐spots and real‐spots, a two‐layer dense autoencoder was employed. The encoder maps low‐pass filtered spectral features ${\tilde{F}}_{s}$ to 512‐dimensional embeddings ${\tilde{F}}_{s}^{*}$, using a linear layer with *LeakyReLU* activation, where s∈{pseudo,real}. The decoder reconstructs the spectral features ${\tilde{F}}_{s}^{r}$ through a dropout layer followed by a linear layer with *LeakyReLU* activation. The autoencoder is trained to maximize similarity between input and reconstructed features, with reconstruction loss defined as:

$$L_{ae}=\sum_{s}|\left|{\tilde{F}}_{s}-{\tilde{F}}_{s}^{r}\right|{|}^{2}$$

This yields embeddings: ${\tilde{F}}_{pseudo}^{*}\in R^{n_{1}\times 512}$ and ${\tilde{F}}_{real}^{*}\in R^{n_{2}\times 512}$.

### 4.7 Cell Type Composition Prediction

The pseudo‐spot cell type compositions are fully known, as each pseudo‐spot is generated by aggregating selected single cells from scRNA‐seq data. Let $Y_{pseudo}\in R^{n_{1}\times n_{ct}}$ denote the ground‐truth cell type proportion matrix for pseudo‐spots, where *n_ct_* is the number of distinct cell types. The goal is to predict the corresponding cell type proportions in real spatial spots, represented as ${\hat{Y}}_{real}\in R^{n_{2}\times n_{ct}}$, by learning the mapping from the low‐dimensional embeddings of pseudo‐spots ${\tilde{F}}_{pseudo}^{*}$ to $Y_{pseudo}$.

To ensure valid compositional predictions, the rows of both $Y_{pseudo}$ and ${\hat{Y}}_{real}$ are constrained to sum to one. The prediction network is a multilayer perceptron (*MLP*) that transforms the encoder‐derived embeddings through a 512‐dimensional hidden layer with *LeakyReLU* activation and dropout for regularization. The hidden representations are then projected to the target dimension *n_ct_* and passed through a *Softmax* function to produce the predicted cell type proportions:

$${\hat{Y}}_{pseudo}=MLP\left({\tilde{F}}_{pseudo}^{*}\right)$$

The model is trained by minimizing the mean squared error (*MSE*) between predicted and true proportions:

$$L_{\mathrm{MSE}}=∥Y_{\mathrm{pseu}\mathrm{do}}-{\hat{Y}}_{\mathrm{pseu}\mathrm{do}}{∥}^{2}$$

To control biologically plausible sparsity in predicted compositions, Hoyer's sparsity regularization [99] is incorporated. For the *i*‐th pseudo‐spot, let ${\hat{y}}_{pseudo}^{i}\in R^{n_{ct}}$ and $y_{\mathrm{pseu}\mathrm{do}}^{i}\in R^{n_{\mathrm{ct}}}$ denote the ground‐truth and predicted cell type vectors, respectively. Sparsity is defined as:

$$\mathrm{spar}\mathrm{sity}\left({\hat{Y}}_{\mathrm{pseu}\mathrm{do}}\right)=\sum_{i}\frac{\sqrt{t}-∥{\hat{y}}_{\mathrm{pseu}\mathrm{do}}^{i}{∥}_{1}/∥{\hat{y}}_{\mathrm{pseu}\mathrm{do}}^{i}{∥}_{2}}{\sqrt{t}-1}$$

where ∥ · ∥_1_ and ∥ · ∥_2_ denote the L1‐norms and L2‐norms, respectively. This term acts as a sparsity regularization on the predicted cell‐type compositions, allowing the users to control the degree of sparsity.

The final loss function integrates both prediction accuracy and sparsity regularization:

$$L_{pred}=L_{MSE}+w\times sparsity\left({\hat{Y}}_{pseudo}\right)$$

where *w* is the regularization coefficient to control sparsity. This design ensures that the model generates accurate, interpretable cell type proportions while maintaining the expected sparse structure of cell type distributions in each spatial spot, consistent with biological constraints.

### 4.8 Model Training

Model training proceeds in two stages. Initially, the autoencoder is pretrained using both pseudo‐spots and real‐spots by minimizing $L_{ae}$ with the Adam optimizer to obtain latent embeddings. In subsequent iterations, only pseudo‐spots are used to optimize $L_{pred}$, focusing on accurate prediction of cell type proportions. The learning rate is progressively decayed over iterations to facilitate convergence. Denoting the initial learning rate as *lr*_0_ (0.0005, default), the learning rate at the *p*‐th iteration is set as $lr^{\left(p\right)}=lr_{0}\times \left(10-\min\left(9,\;p\right)/10\right)$.

### 4.9 Performance Evaluation

#### 4.9.1 Generation of Simulated Datasets

To rigorously assess deconvolution performance, we generated simulated spatial transcriptomics datasets with known ground‐truth cell type compositions. A mouse brain Slide‐seqV2 dataset, which approximates single‐cell resolution, served as the basis for simulation. Each Slide‐seqV2 spot was replaced by the most similar scRNA‐seq cell based on expression similarity. The tissue was partitioned into spatial grids, and cells within each grid were aggregated to form synthetic spots. Five simulation scenarios were designed to evaluate different aspects of performance. In Scenario 1, the grid size was set to 55×55, and in Scenario 2, it was increased to 100 × 100 to assess the effect of spatial resolution on deconvolution accuracy. Scenario 3 evaluated the robustness to incomplete reference data by removing the low‐abundance Endothelial‐Fit1 cell type from the scRNA‐seq reference. Scenario 4 assessed performance when the reference contained an extra cell type that is not present in the tissue by adding Choroid_Plexus_Ttr to the reference. Scenario 5 introduced a simulation setting where approximately 5% of cells in the reference dataset were randomly assigned incorrect cell type labels. In MERFISH simulation, Bregma ‐0.04 section was partitioned into 55 × 55 grid, with the Bregma 0.11 section serving as the reference. In SeekSpace simulation, WH1035 section was partitioned into 400 × 400 grid with WH938 section serving as the reference.

#### 4.9.2 Evaluation Metrics

Deconvolution performance was evaluated using four complementary metrics: Root Mean Squared Error (*RMSE*), Mean Absolute Error (*MAE*), Jensen–Shannon Divergence (*JSD*), and Pearson Correlation Coefficient (*PCC*). Evaluation was performed at two levels: (i) spot‐level, comparing predicted versus ground‐truth cell type proportions for each spot, and (ii) cell‐type‐level, comparing the spatial distribution patterns for each cell type. Spot‐level metrics capture local prediction accuracy, whereas cell‐type‐level metrics reflect the preservation of global spatial organization.

For a given spot, let $y=(y_{1},\;y_{2},\cdots ,y_{t})$ 7and $\hat{y}=({\hat{y}}_{1},\;{\hat{y}}_{2},\text{…},{\hat{y}}_{t}$) denote the true and predicted cell type proportion vectors, respectively. The metrics are defined as follows:

Root Mean Squared Error (*RMSE*):

$$\mathrm{RMSE}\left(y,\hat{y}\right)=\sqrt{\frac{1}{t}\sum_{k}{\left(y_{k}-{\hat{y}}_{k}\right)}^{2}}$$

Mean Absolute Error (*MAE*):

$$\mathrm{MAE}\left(y,\hat{y}\right)=\frac{1}{t}\sum_{k}\left|y_{k}-{\hat{y}}_{k}\right|$$

Jensen–Shannon Divergence (*JSD*):

$$\mathrm{JSD}\left(y∥\hat{y}\right)=\frac{1}{2}D_{\mathrm{KL}}\left(y∥m\right)+\frac{1}{2}D_{\mathrm{KL}}\left(\hat{y}∥m\right),m=\frac{1}{2}\left(y+\hat{y}\right)$$

where *D_KL_* denotes the Kullback–Leibler (*KL*) divergence.

Pearson Correlation Coefficient (*PCC*):

$$\mathrm{PCC}\left(y,\hat{y}\right)=\frac{\sum_{k}\left(y_{k}-\bar{y}\right)({\hat{y}}_{k}-\bar{\hat{y}})}{\sqrt{\sum_{k}{\left(y_{k}-\bar{y}\right)}^{2}}\sqrt{\sum_{k}{\left({\hat{y}}_{k}-\bar{\hat{y}}\right)}^{2}}}$$

The same metrics were computed at the cell‐type level to assess the accuracy of spatial distribution predictions across the tissue. Together, these metrics provide a comprehensive evaluation framework capturing both local accuracy and global spatial fidelity.

### 4.10 Software Configurations

#### 4.10.1 Cell2location (Version 0.1)

According to the tutorial, we conducted Cell2location analysis to deconvolve spatial transcriptomics data using matched single‐cell references. First, scRNA‐seq and spatial datasets were preprocessed: low‐quality cells and genes were filtered, and highly informative genes were selected. Next, a regression model was trained on the single‐cell data to estimate cell type–specific expression profiles. These profiles were then used as references for training the Cell2location model on the spatial dataset. The model was trained with default parameters (e.g., expected cell number per spot, detection alpha). Posterior estimates of cell type abundances were exported and normalized to obtain the relative proportions per spot.

#### 4.10.2 DestVI (Version 0.14.0)

We applied DestVI to deconvolve spatial transcriptomics data using matched single‐cell references based on scvi‐tools according to its tutorials. First, scRNA‐seq and spatial datasets were preprocessed: low‐quality cells and genes were filtered, and highly variable genes were selected. The single‐cell data were used to train a conditional scVI (CondSCVI) model to learn cell type–specific latent representations. The trained model then served as a reference for DestVI, which was applied to the spatial dataset to estimate spot‐level cell type proportions. Training of both CondSCVI and DestVI was performed with default parameters for number of epochs and learning rate. Finally, the estimated cell type proportions for each spot were exported as normalized relative abundances.

#### 4.10.3 Stereoscope (Version 0.14.0)

Following the tutorial in scvi‐tools, first, both scRNA‐seq and spatial datasets were preprocessed: low‐quality genes and cells were filtered, mitochondrial genes were removed, and highly variable genes were selected. The scRNA‐seq data were used to train a *RNAStereoscope* model, which learned cell type–specific expression profiles. The trained model then served as a reference for *SpatialStereoscope*, which was applied to the spatial dataset to estimate spot‐level cell type proportions. Training of both *RNAStereoscope* and *SpatialStereoscope* was performed with default parameters for number of epochs. Finally, the estimated cell type proportions for each spot were exported as normalized relative abundances.

#### 4.10.4 Tangram (Version 1.0.4)

Following the Tangram tutorial, both scRNA‐seq and spatial datasets were first preprocessed: cells with very few samples per cell type were removed, and the top marker genes per cell type were identified using *scanpy.tl.rank_genes_groups*. Shared genes between the scRNA‐seq and spatial datasets were selected for mapping. Tangram was then used to map cell type annotations from single cells to spatial spots (*mode═'clusters'*), and the resulting spot‐level cell type densities were normalized to sum to one. Default parameters were used throughout, and the normalized cell type proportions for each spot were exported for downstream analysis.

#### 4.10.5 RCTD (Version 2.2.1)

We conducted RCTD following its tutorial. Briefly, the scRNA‐seq and spatial datasets were preprocessed to remove cell types with fewer than 25 cells and ensure unique cell type labels. A single‐cell reference object was created from expression counts and cell type annotations, and a *SpatialRNAobject* was generated from spatial counts and coordinates. An RCTD object was created with *create.RCTD* and run in *doublet_mode = ‘full’* with default parameters to estimate normalized cell type proportions at each spatial spot.

#### 4.10.6 SpatialDWLS (Version 4.2.2)

We implemented SpatialDWLS following its tutorial. Briefly, scRNA‐seq and spatial datasets were loaded as Giotto objects and normalized. Highly variable genes were identified and PCA was performed on both datasets. Cell‐type marker genes were identified from the scRNA‐seq data, and a signature matrix was constructed using the mean normalized expression of the top markers per cluster. Using these signatures, SpatialDWLS inferred cell type proportions for each spatial spot with default parameters.

#### 4.10.7 DSTG (Version 0.0.1)

We implemented DSTG following its tutorial. Briefly, scRNA‐seq reference and spatial transcriptomics data were loaded and restricted to shared genes, and scRNA‐seq cell‐type labels were one‐hot encoded. The two datasets were concatenated to form a unified feature matrix. PCA and a k‐nearest neighbor graph were then constructed to build a joint cell–spot adjacency matrix. These features, labels, and graph structure were used as input to the DSTG graph convolutional network, which was trained in a semi‐supervised manner using only scRNA‐seq labels. The trained model generated *softmax* outputs for spatial spots, which were interpreted as cell‐type proportions per spot.

#### 4.10.8 SONAR (Version 1.1.0)

We implemented SONAR following its tutorial. Spatial transcriptome deconvolution was performed using the SONAR package implemented in R. Raw count matrices were extracted from the single‐cell RNA‐seq reference dataset and spatial transcriptome dataset, followed by integer rounding and filtering of low‐expression spots. Shared highly variable genes between the two datasets were retained as input features. Input data were preprocessed using the *SONAR.preprocess* function. The preprocessed data were formatted and transferred to the MATLAB core computing environment via the *SONAR.deliver* function. Deconvolution analysis was executed using the *SONAR.deconvolute* function with the core MATLAB script *SONAR_main.m*. MATLAB‐formatted deconvolution outputs.

#### 4.10.9 SPOTlight (Version 1.10.0)

We implemented SPOTlight following its tutorial. Briefly, single‐cell RNA‐seq and spatial datasets were loaded into *SingleCellExperiment* and *SpatialExperiment* objects, respectively. The scRNA‐seq data were normalized. Then highly variable genes were implemented, and marker genes were selected for each cell type. These marker genes, together with the *SingleCellExperiment* and *SpatialExperiment* objects, were used as input for SPOTlight deconvolution with default parameters.

#### 4.10.10 STdGCN (Version 0.0.1)

We implemented STdGCN following its tutorial. Briefly, scRNA‐seq and spatial transcriptomics data were loaded and normalized. Marker genes were identified from the single‐cell reference using *scanpy.tl.rank_genes_groups*. Synthetic pseudo‐spots were simulated from scRNA‐seq data and real and pseudo‐spots were normalized and integrated. A multi‐part expression graph was constructed using mutual nearest neighbors (MNN) with cosine distance and a spatial graph was built with a soft linkage rule and distance threshold = 2. The integrated graph and features were used as input for STdGCN deconvolution with default parameters.

#### 4.10.11 CLPLS

We implemented CLPLS following its tutorial. Briefly, scRNA‐seq reference and spatial transcriptomics data were loaded and normalized into PCA space. For spatial transcriptomics data, a spatial adjacency matrix, an expression‐based neighborhood graph, and contrastive learning labels were generated. For scRNA‐seq data, cell‐type labels and a cell type restricted adjacency matrix were constructed. These graph structures and labels were then used as input for CLPLS's graph‐based representation learning model. Learned embeddings for single‐cell and spatial data were projected with PLS regression (PLSR) using 5 components to infer cell‐type proportions per spatial spot.

#### 4.10.12 STAID

For each spatial transcriptomics dataset, we used a matched scRNA‐seq reference with annotated cell types. STAID generates pseudo‐spots and iteratively updates cell type proportions. For the 100 × 100 spatial datasets, the number of cells per pseudo‐spot was randomly sampled from 1 to 12, while for the 55×55 datasets, it was sampled from 1 to 8. In our implementation, each iteration used 5,000 pseudo‐spots, a learning rate of 0.005, 20 iteration steps, a sparsity weight of 0.00001, and a batch size of 128.

### 4.11 Cell Type Co‐Localization Analysis

Spatial co‐localization between cell types was quantified by computing Spearman's rank correlation coefficients between their predicted spot‐level proportions across the tissue section. Positive coefficients indicate spatially concordant distribution patterns, whereas negative coefficients reflect mutually exclusive spatial organization.

### 4.12 The Spatial Correlation Between Cell Types and Genes

To characterize spatial associations between gene expression and cellular composition, we calculated Spearman's rank correlation coefficients between predicted cell‐type proportions and gene expression levels across all spots. Gene–cell‐type pairs exceeding a predefined correlation threshold were retained as spatially concordant, thereby indicating shared anatomical or functional localization.

### 4.13 Gene Ontology Enrichment Analysis

For each correlated gene set, we performed Gene Ontology (GO) enrichment analysis using the gseapy tool [100] based on the *GO_Biological_Process_2023* database. Multiple testing correction was applied using the Benjamini‐Hochberg method and GO terms with adjusted *p*‐value < 0.05 were retained. These significantly enriched biological terms were then used for functional interpretation of the corresponding gene sets.

### 4.14 TLS‐Like Region Scoring in Crohn's Disease Tissues

To characterize TLS‐like immune niches, we adopted TLS‐associated gene signatures reported previously [85]. We computed a TLS score for each spatial spot by averaging the log‐transformed expression levels of these TLS‐related genes. Spots with TLS scores above the 90th percentile were defined as TLS‐like regions, representing areas with coordinated upregulation of TLS‐associated genes. This approach enables the identification of regions with B/T cell enrichment and other molecular features reminiscent of TLS, consistent with known biology.

## Author Contributions

B.L. conceptualized and supervised the project. J.L. designed and implemented the method. J.L., S.S., and Z.L. carried out the simulations and benchmarking. J.L., B.L., X.L., and Y.W. analyzed and interpreted the results in the case studies. All authors have read and approved the final manuscript.

## Ethics Approval Statement

The authors have nothing to report.

## Consent

The authors have nothing to report.

## Conflicts of Interest

The authors declare no conflicts of interest.

## Code Availability

The STAID algorithm is implemented in Python and is publicly available on GitHub (https://github.com/jxLiu‐bio/STAID) under MIT license.

## Supporting information




**Supporting File**: advs75607‐sup‐0001‐SuppMat.docx.

## Data Availability

All datasets used in this study are publicly available and this study did not generate any new data. The mouse brain Slide‐seqV2 dataset Puck_191204_01 for simulation dataset generation is available for download at the Broad Institute Single Cell Portal at https://singlecell.broadinstitute.org/single_cell/study/SCP815/highly‐sensitive‐spatial‐transcriptomics‐at‐near‐cellular‐resolution‐with‐slide‐seqv2#study‐summary [55]. The human breast cancer Visium datasets are available at https://doi.org/10.5281/zenodo.4739739 and match human breast cancer scRNA‐seq reference datasets are available through the Gene Expression Omnibus under accession number GSE176078 [65]. The human embryonic limb Visium datasets and matched scRNA‐seq reference are available for download at https://limb‐dev.cellgeni.sanger.ac.uk [73]. The MERFISH datasets are available at https://doi.org/10.5061/dryad.8t8s248 [101]. The SeekSpace datasets can be downloaded at https://ngdc.cncb.ac.cn/omix under accession no. OMIX016242 [102]. The human Crohn's disease Visium datasets and matched scRNA‐seq datasets are available at https://singlecell.broadinstitute.org/single_cell/study/SCP2959/human‐cd‐fibrosis‐study‐using‐single‐cell‐and‐spatial‐data [19].
