# Supplementary material for: STAID: A Self‐Refining Deep Learning Framework for Spatial Cell‐Type Deconvolution with Biologically Informed Modeling
Source: Adv Sci (Weinh). 2026 May 10;13(43):e75607. doi: 10.1002/advs.75607 (PMC13335853; doi:10.1002/advs.75607)
Supplement: Supplementary file 1 — Supporting File: advs75607‐sup‐0001‐SuppMat.docx. [file ADVS-13-e75607-s001.docx]

**Supplementary Information**

**Supplementary Figures**


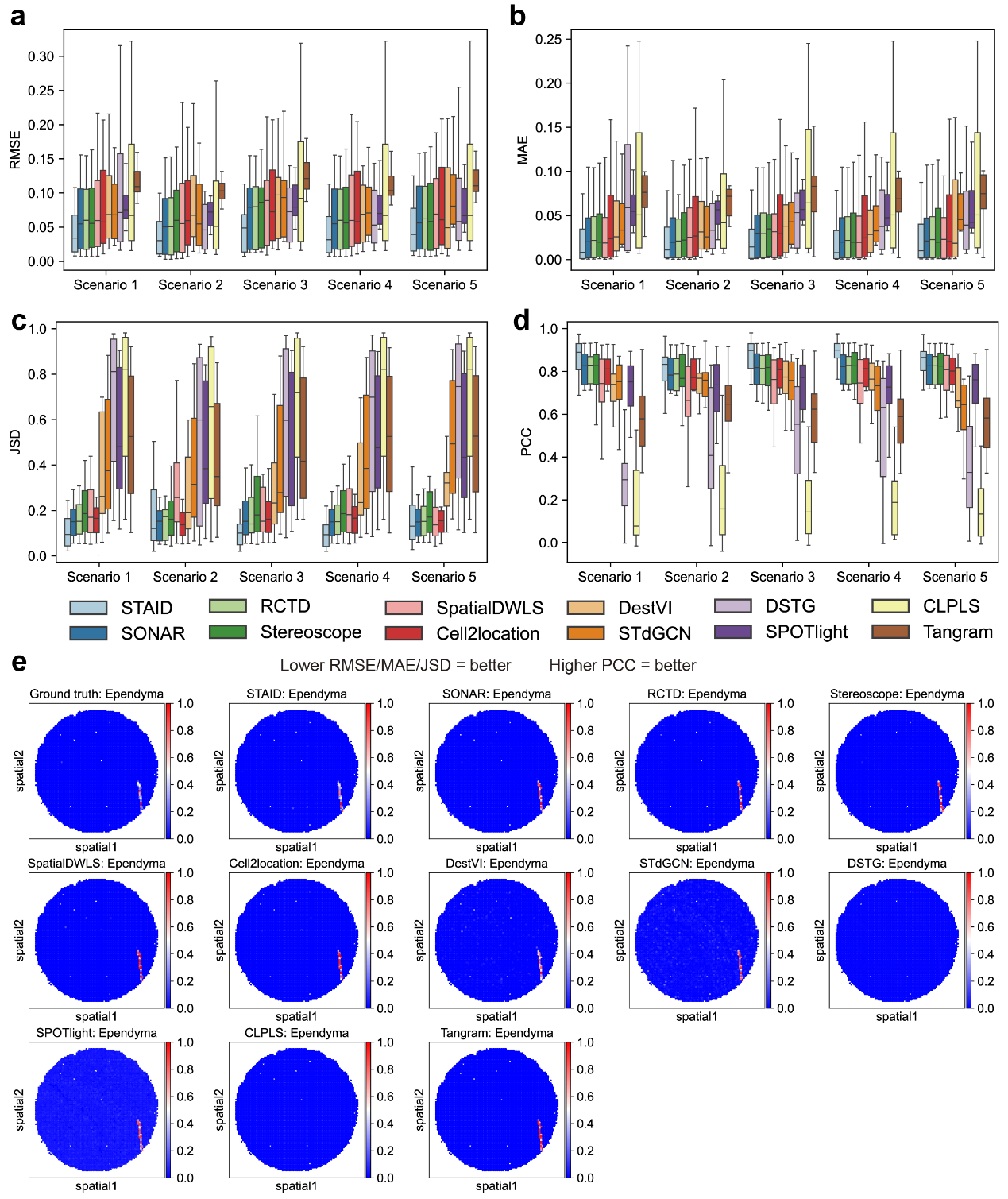


**Supplementary Fig. 1 | STAID outperforms existing methods in simulated scenarios and improves through iterative refinement at cell-type level. a–d**, Performance comparison at cell-type level across four evaluation metrics: *RMSE* (a), *MAE* (b), *JSD* (c), and *PCC* (d). **e**, Predicted spatial distribution of Ependyma across different methods with the ground truth.


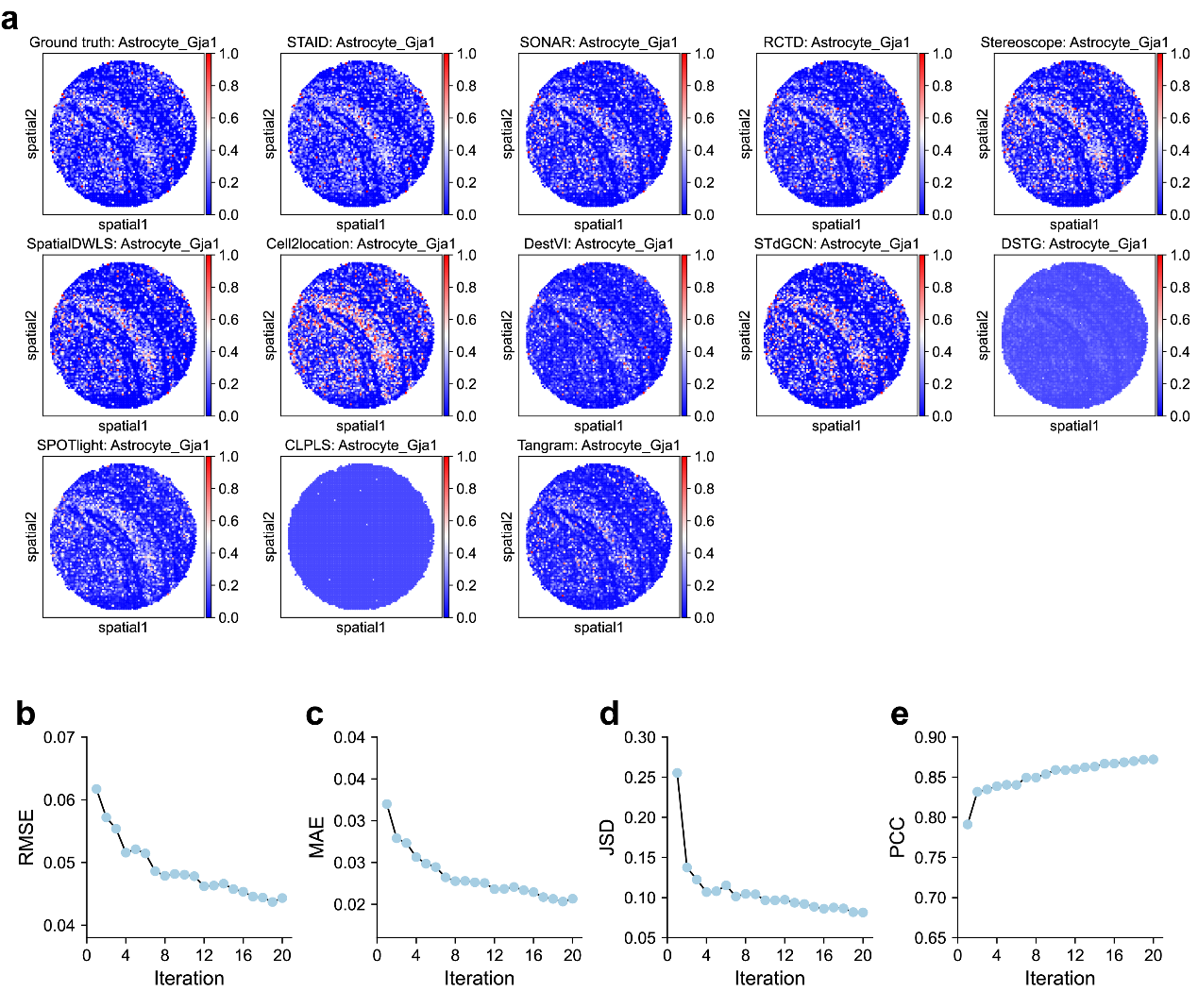


**Supplementary Fig. 2 | Cell type distribution patterns comparison among different methods. a**, Predicted spatial distribution of Astrocyte_Gja1 across different methods with the ground truth. **b–e**, Evaluation metrics (*RMSE*, *MAE*, *JSD*, and *PCC*) at cell type level across iterations. The predictive performance progressively improves as the number of iterations increases.

**
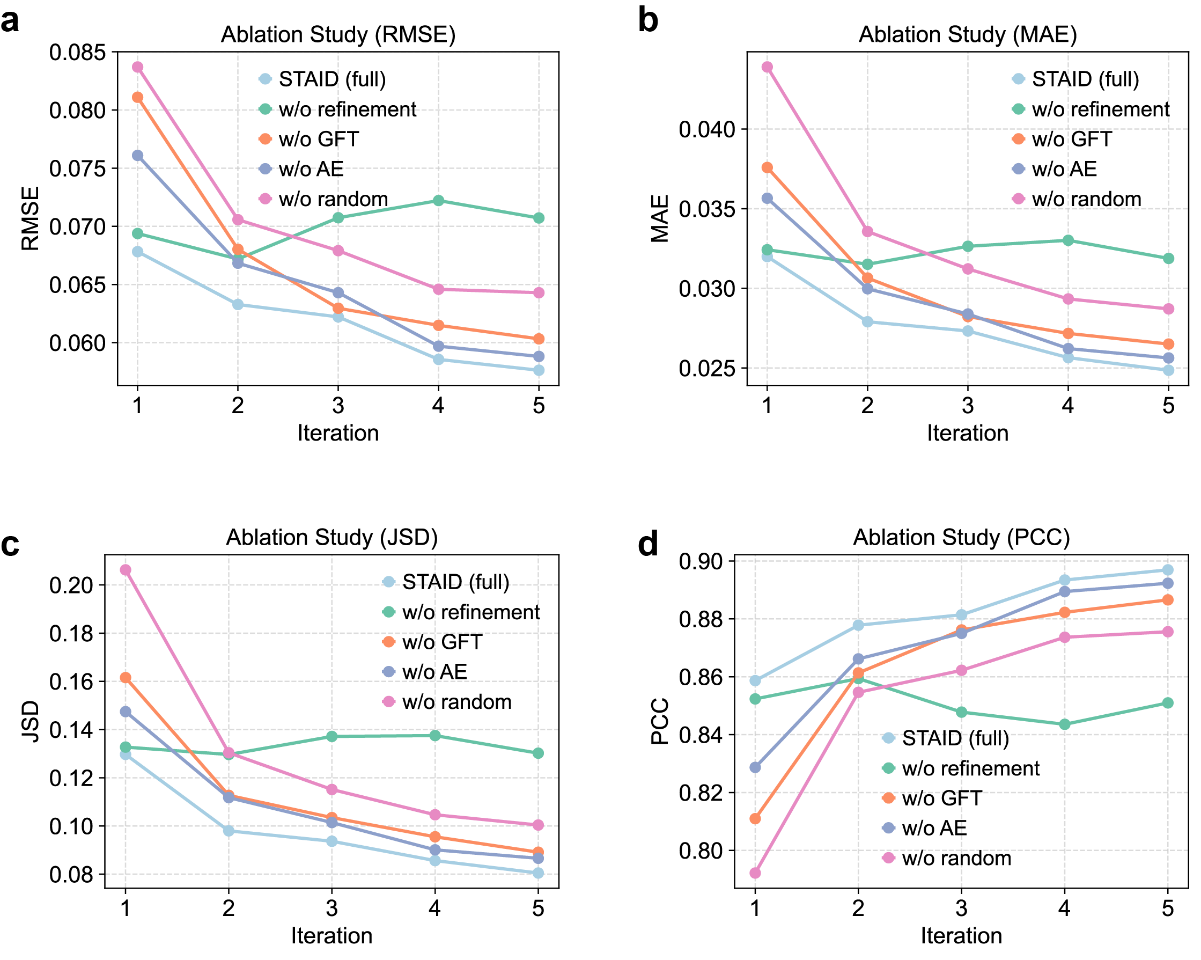
**

**Supplementary Fig. 3** **| Ablation study of the STAID framework.** Performance comparison across iterations between the full STAID model and four ablated variants: without pseudo-spot refinement (w/o refinement), graph Fourier transform (w/o GFT), the autoencoder (w/o AE), and random supplementation (w/o random). **a–d**, Evaluation metrics include: (a) root mean squared error (*RMSE*), (b) mean absolute error (*MAE*), (c) Jensen–Shannon divergence (*JSD*), and (d) Pearson correlation coefficient (*PCC*).

**
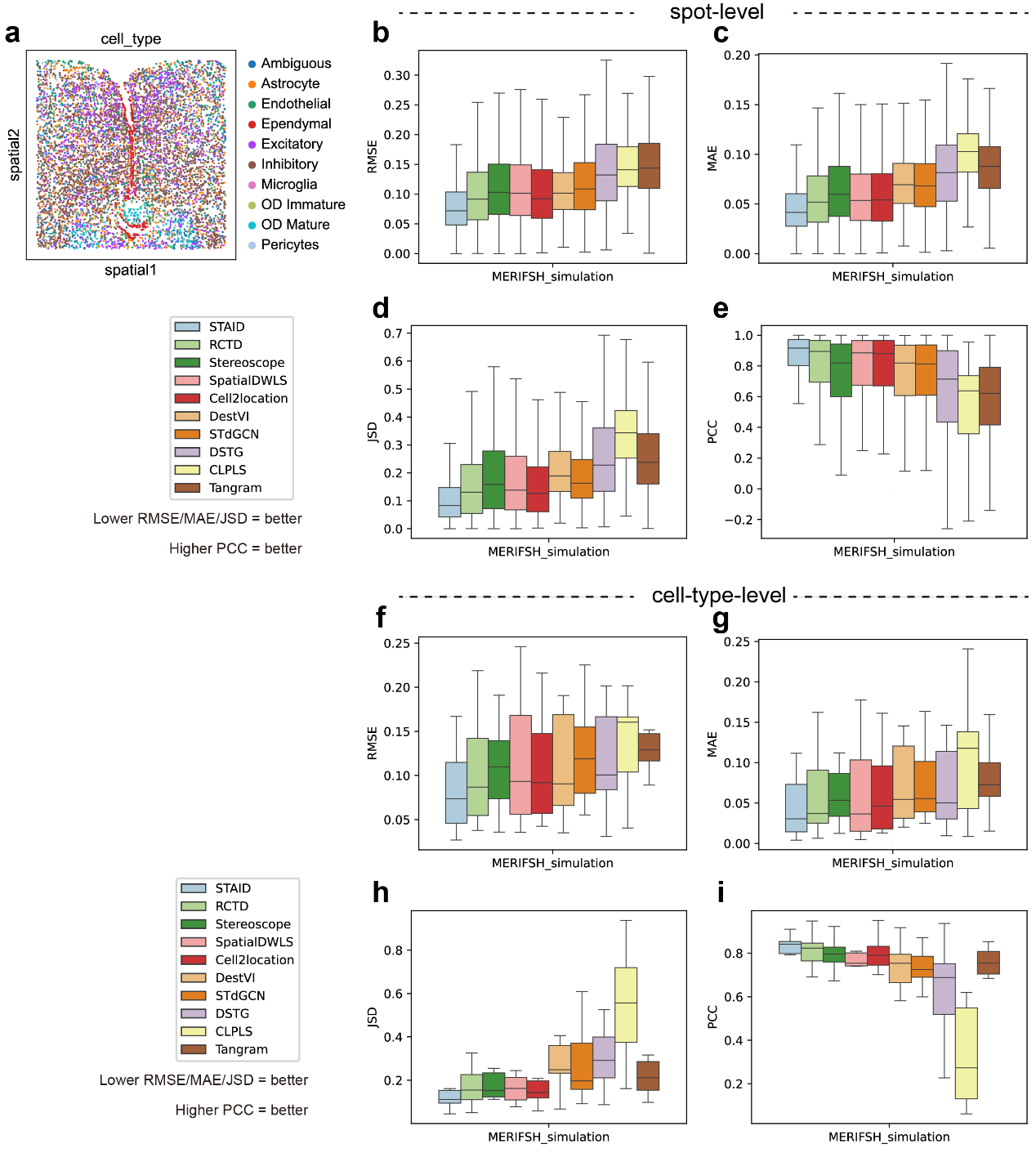
**

**Supplementary Fig. 4 | Benchmarking of STAID against other deconvolution methods on MERFISH simulation data. a**, Spatial distribution of annotated cell types in MERFISH. **b–e**, Quantitative comparison of spot-level performance across methods using four metrics: *RMSE* (b), *MAE* (c), *JSD* (d), and *PCC* (e), where lower values indicate better reconstruction accuracy for *RMSE*, *MAE*, and *JSD*, and higher values indicate better correlation for *PCC*. **f–i**, Quantitative comparison of cell-type-level performance across methods using the same four metrics: *RMSE* (f), *MAE* (g), *JSD* (h), and *PCC* (i).

**
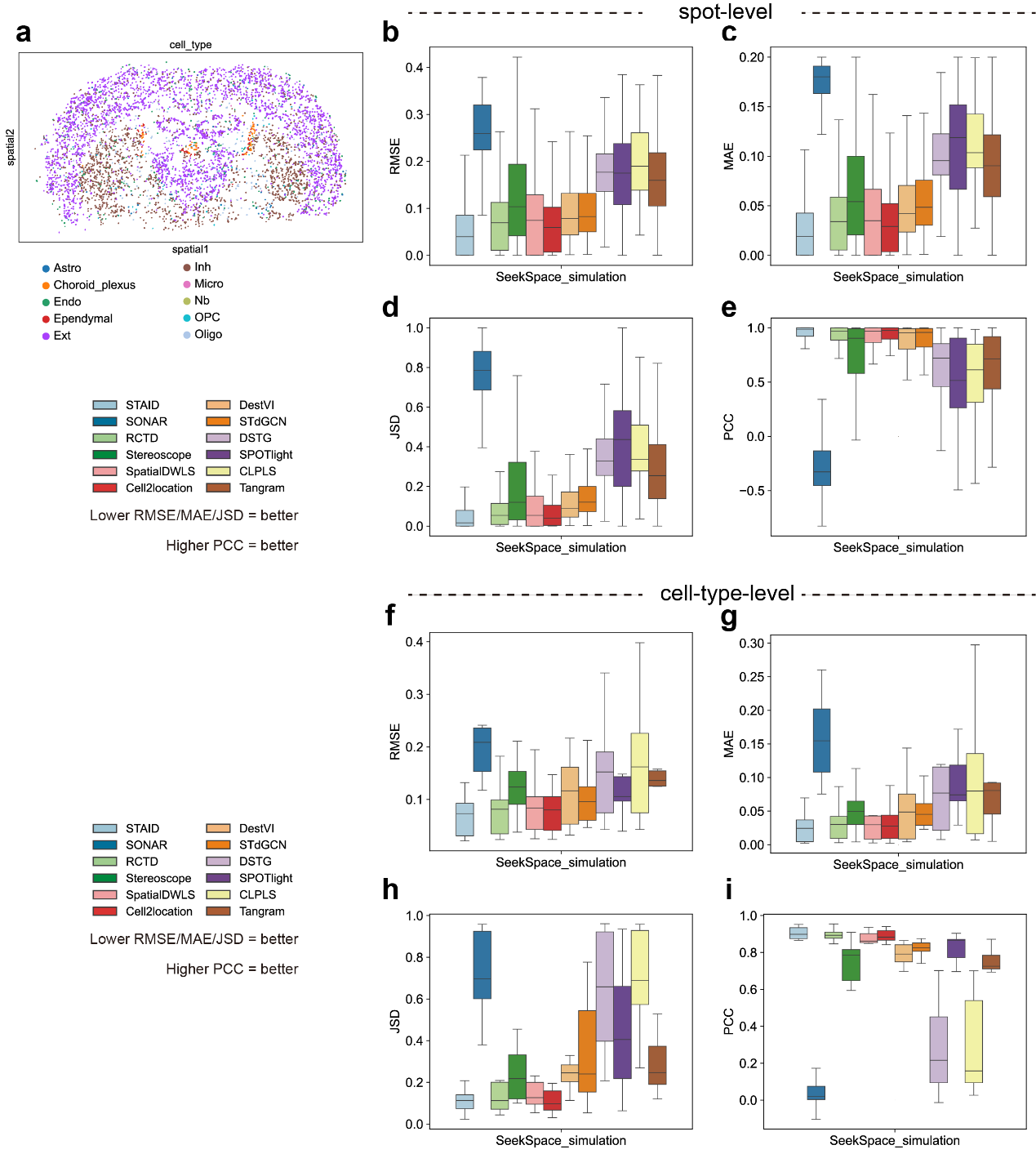
**

**Supplementary Fig. 5 | Benchmarking of STAID against other deconvolution methods on SeekSpace simulation data. a**, Spatial distribution of annotated cell types in SeekSpace. **b–e**, Quantitative comparison of spot-level performance across methods using four metrics: *RMSE* (b), *MAE* (c), *JSD* (d), and *PCC* (e), where lower values indicate better reconstruction accuracy for *RMSE*, *MAE*, and *JSD*, and higher values indicate better correlation for PCC. **f–i**, Quantitative comparison of cell-type-level performance across methods using the same four metrics: *RMSE* (f), *MAE* (g), *JSD* (h), and *PCC* (i).


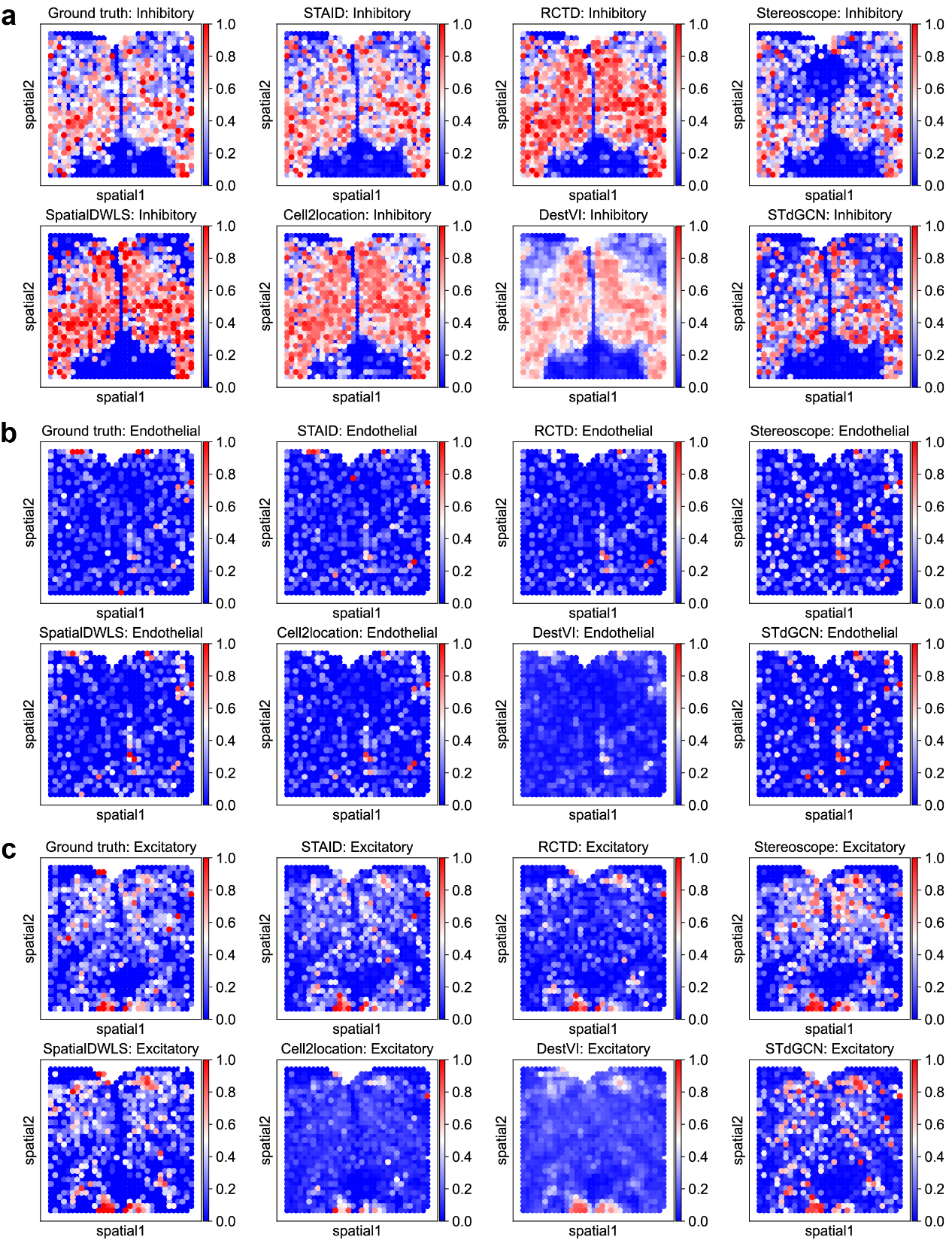


**Supplementary Fig. 6 | Comparison of spatial cell-type distributions across deconvolution methods on MERFISH simulation data. a**, Predicted spatial distributions of the Inhibitory neurons by STAID and competing deconvolution methods, compared with the ground truth. **b**, Predicted spatial distributions of the Endothelial cell type by STAID and competing methods, overlaid with the ground truth. **c**, Predicted spatial distributions of the Excitatory neurons by STAID and competing methods, compared to the ground truth.

**
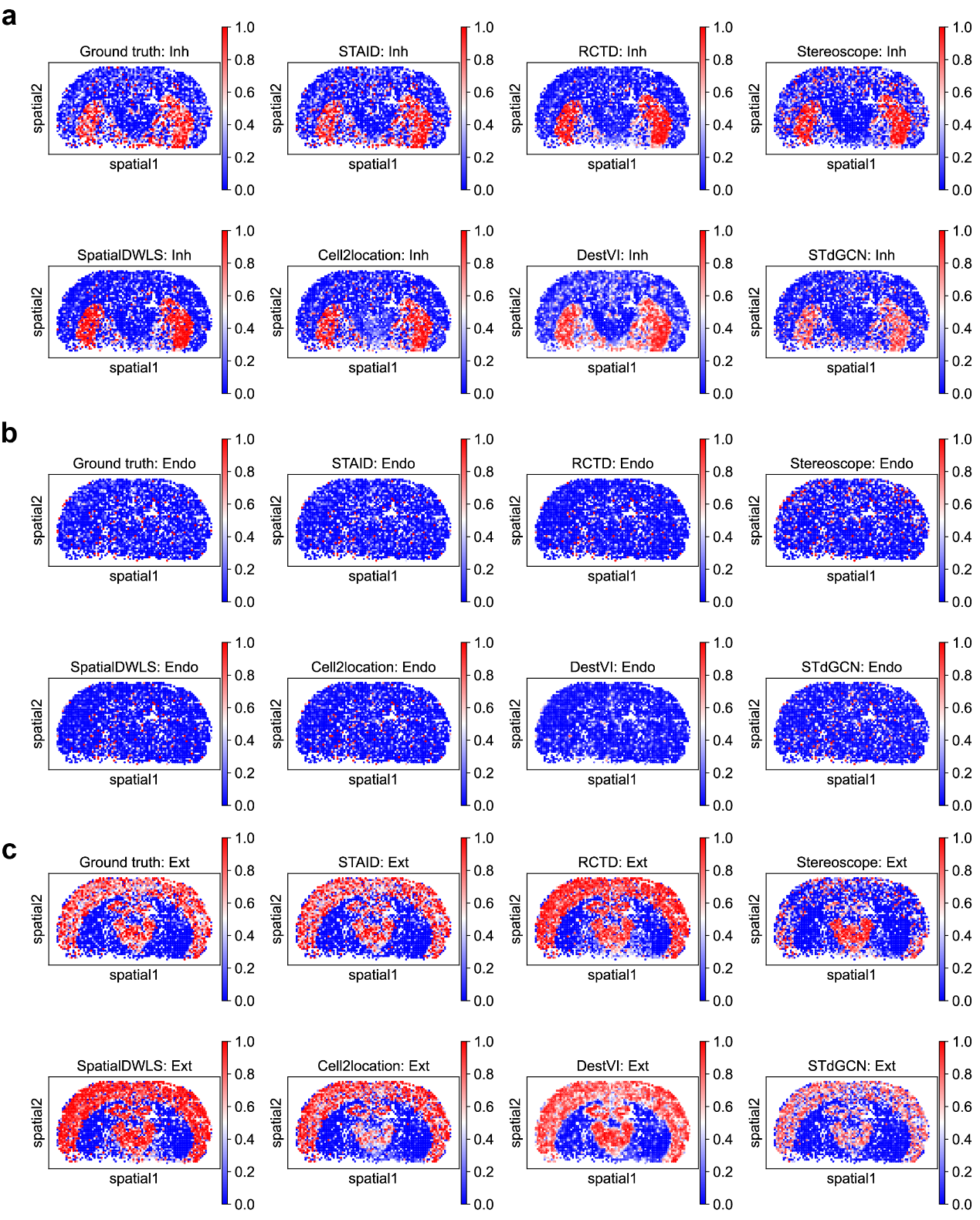
**

**Supplementary Fig. 7 |** **Comparison of spatial cell-type distributions across deconvolution methods on SeekSpace simulation data. a**, Predicted spatial distributions of the Inhibitory neurons (Inh) type by STAID and competing deconvolution methods, compared with the ground truth. **b**, Predicted spatial distributions of the Endothelial (Endo) cell type by STAID and competing methods, overlaid with the ground truth. **c**, Predicted spatial distributions of the Excitatory neurons (Ext) by STAID and competing methods, compared to the ground truth.

**
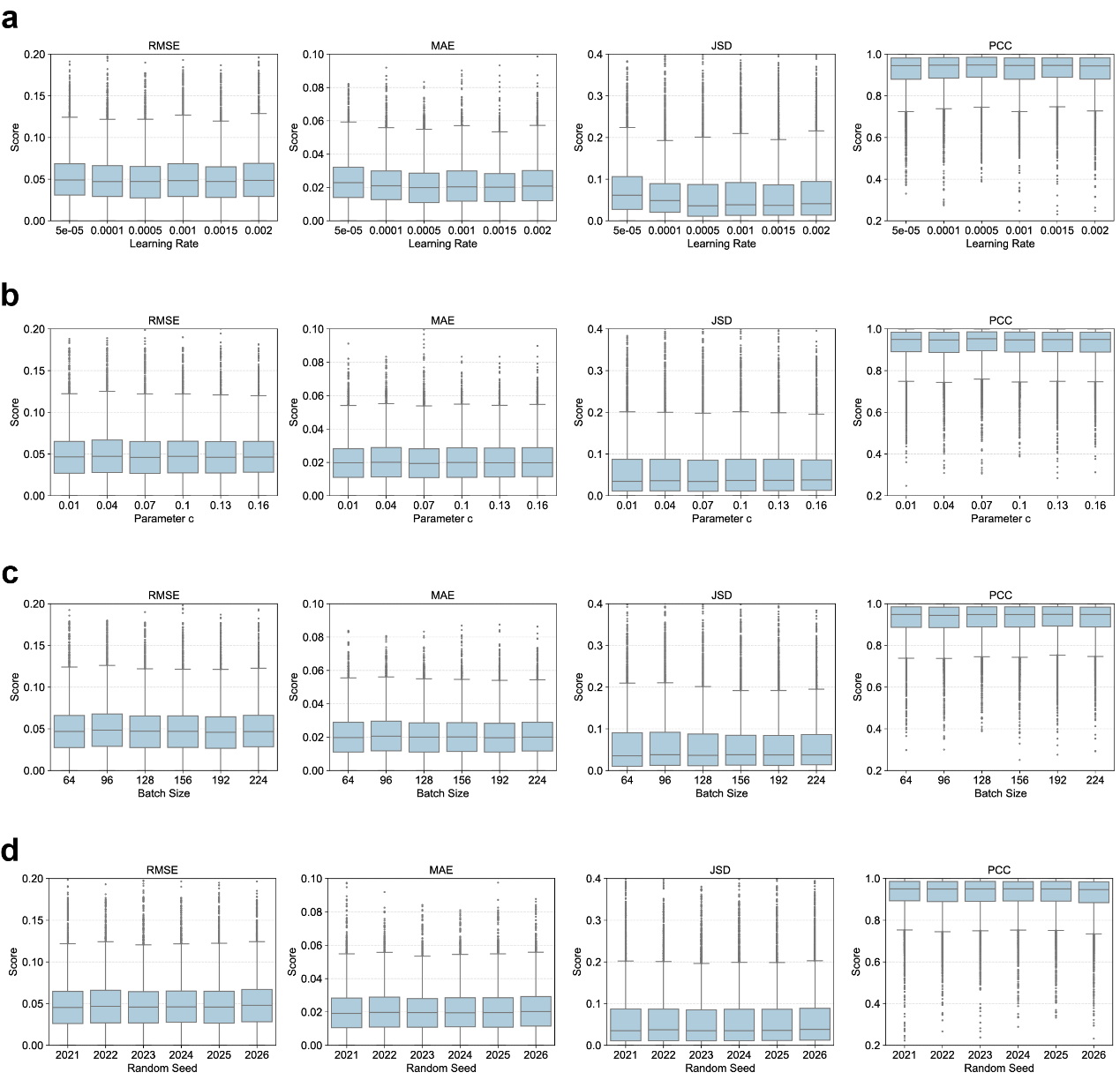
**

**Supplementary Fig. 8 | Sensitivity analysis of STAID across key hyperparameters. a**, Performance of STAID with different learning rates. Four evaluation metrics (*MAE*, *RMSE*, *PCC*, *JSD*) are reported, with boxplots summarizing results across replicates for learning rates ranging from 5e-5 to 0.002. **b**, Performance of STAID with different low-pass filtering parameter *c*. Results are shown across values from 0.01 to 0.16. **c**, Performance of STAID with different batch sizes. Results are reported for batch sizes ranging from 64 to 224. **d**, Performance of STAID with different random seeds. Boxplots summarize results across seeds 2021–2026.


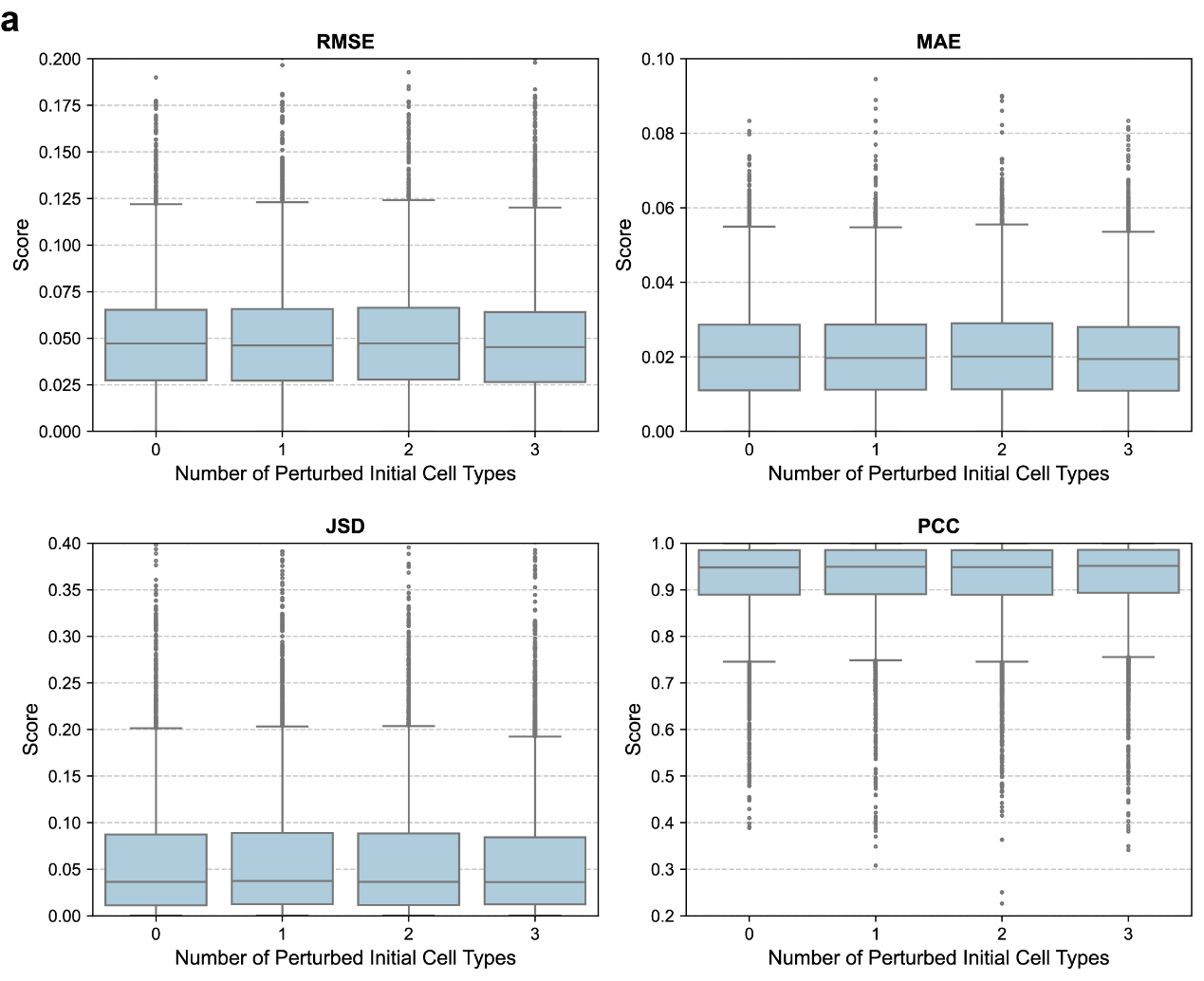


**Supplementary Fig. 9 | Performance variation of STAID with perturbed initial cell-type enrichment estimates. a**, Four evaluation metrics (*RMSE*, *MAE*, *JSD*, *PCC*) are reported under different numbers of perturbed enriched initial cell types. STAID shows stable performance across perturbation levels.


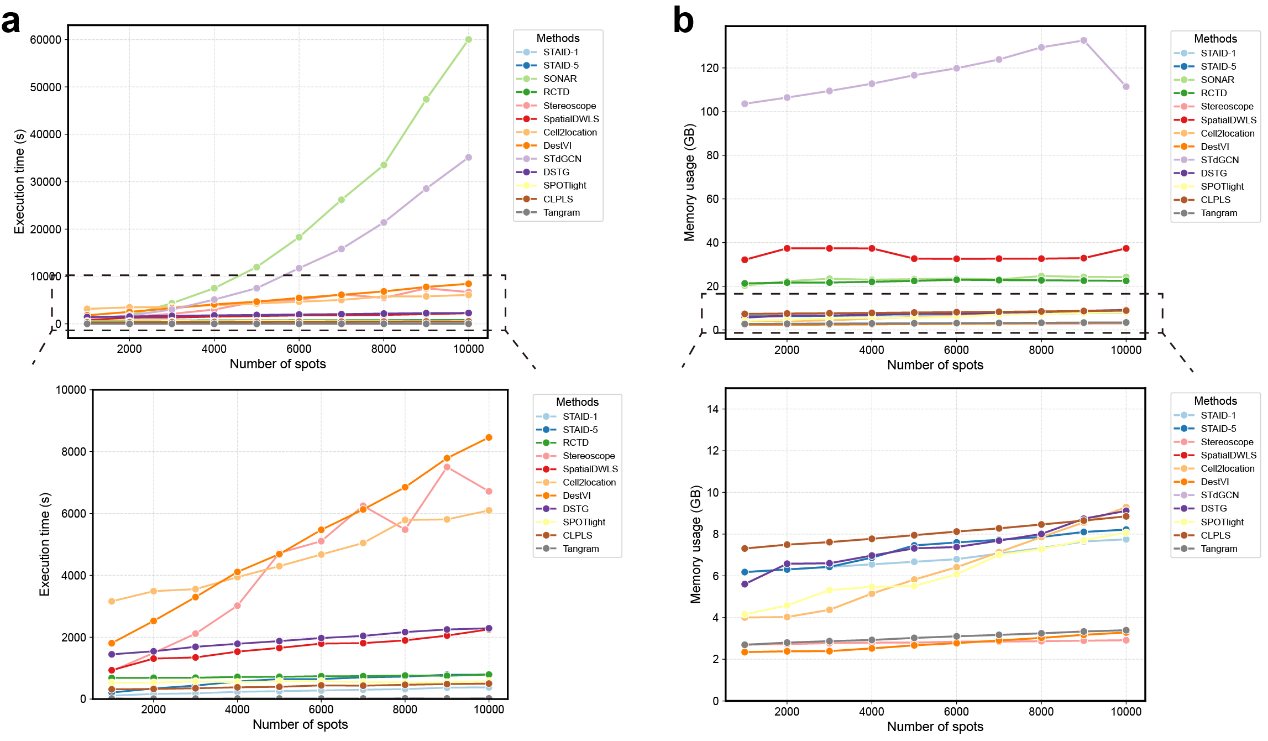


**Supplementary Fig. 10 | Running time and memory usage.** Running time and memory usage were measured on a system running CentOS, equipped with a 24-core CPU and an NVIDIA A100 GPU (40 GB). Execution time and memory usage were recorded for each method. **a**, Runtime comparison across methods with increasing numbers of spatial spots (1,000–10,000). Full results include all methods (top), with a subset shown for clarity (bottom). Execution time (seconds) is reported for STAID (1-iteration and 5-iteration variants) and baselines. **b**, Memory usage (GB) under increasing dataset sizes. Full results include all methods (top), with a subset shown for clarity (bottom).


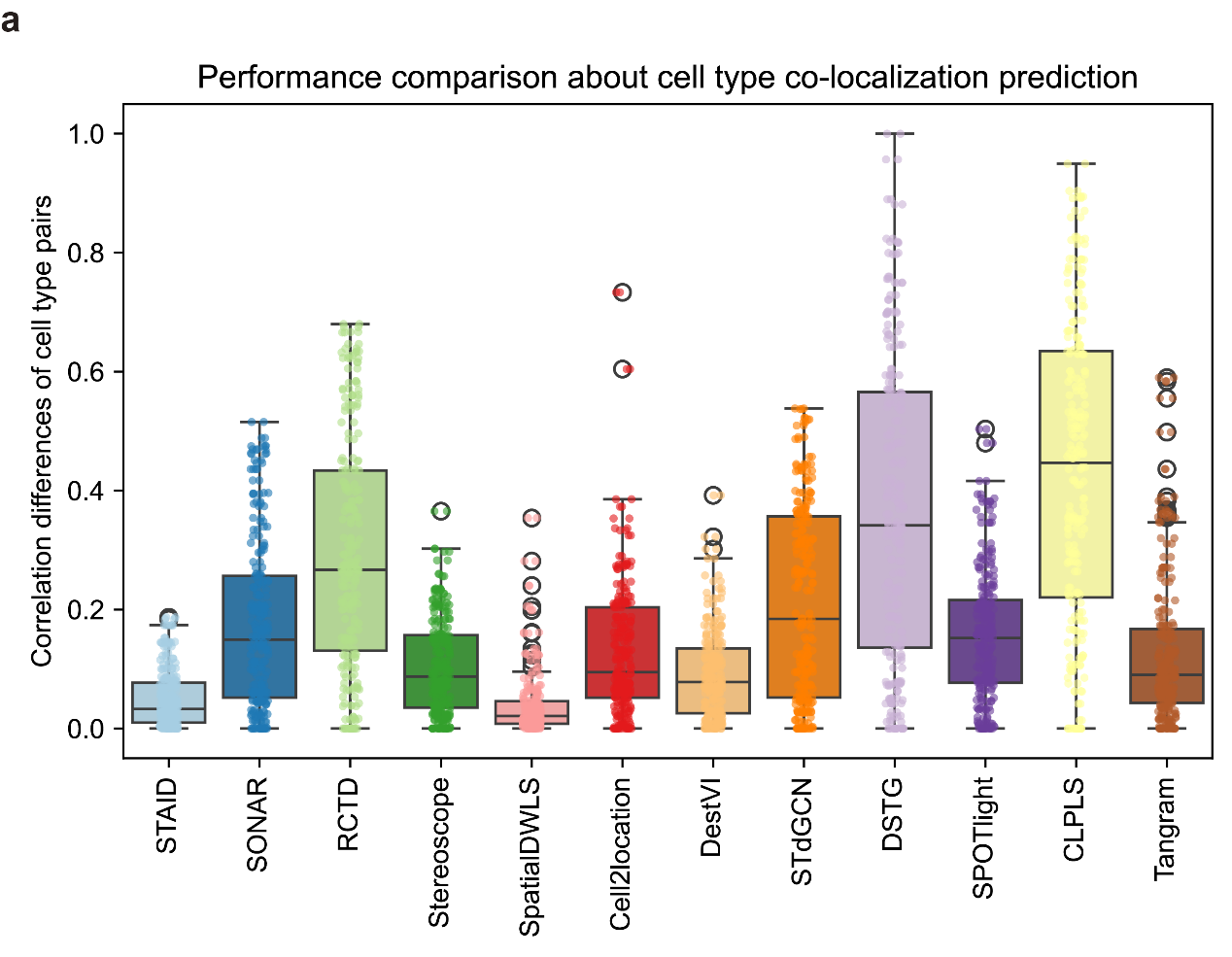


**Supplementary Fig. 11 | Comparison of cell-type co-localization patterns across methods. a**, Pairwise Spearman's rank correlation coefficients between cell type pairs were computed from predicted cell-type compositions and compared with the ground truth. The box plot shows the differences of Spearman's rank correlation coefficients inferred by computational methods relative to the true correlations. STAID and SpatialDWLS exhibits better performance.

**
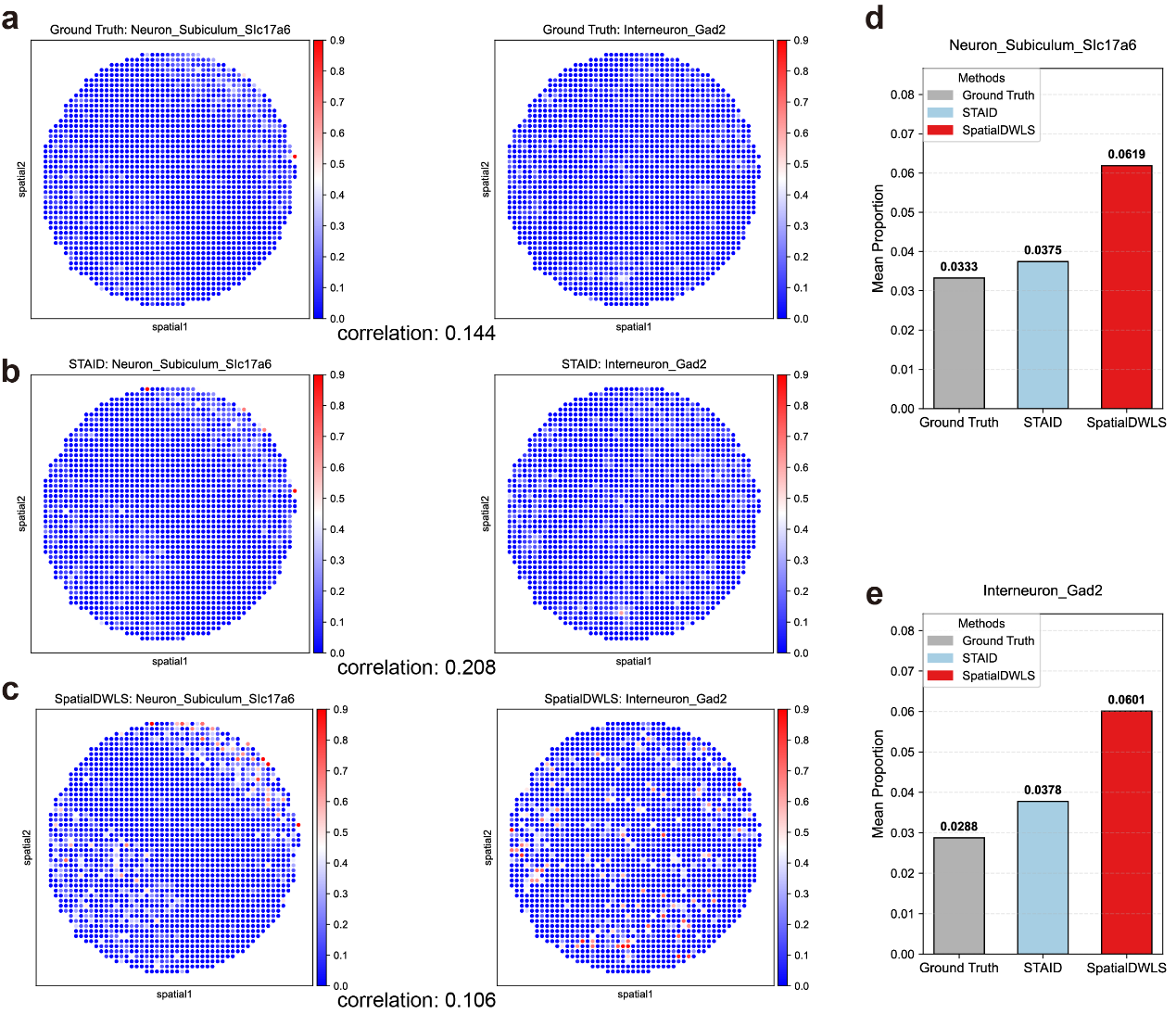
**

**Supplementary Fig. 12 | Comparison of spatial distributions and cell-type proportions. a–c,** Spatial distributions of Neuron_Subiculum_Slc17a6 and Interneuron_Gad2. (a) Ground truth (correlation coefficient = 0.144), (b) STAID prediction (correlation coefficient = 0.208), and (c) SpatialDWLS prediction (correlation coefficient = 0.106). **d–e**, Proportions of the two cell types across ground truth, STAID, and SpatialDWLS. (d) Neuron_Subiculum_Slc17a6, (e) Interneuron_Gad2.


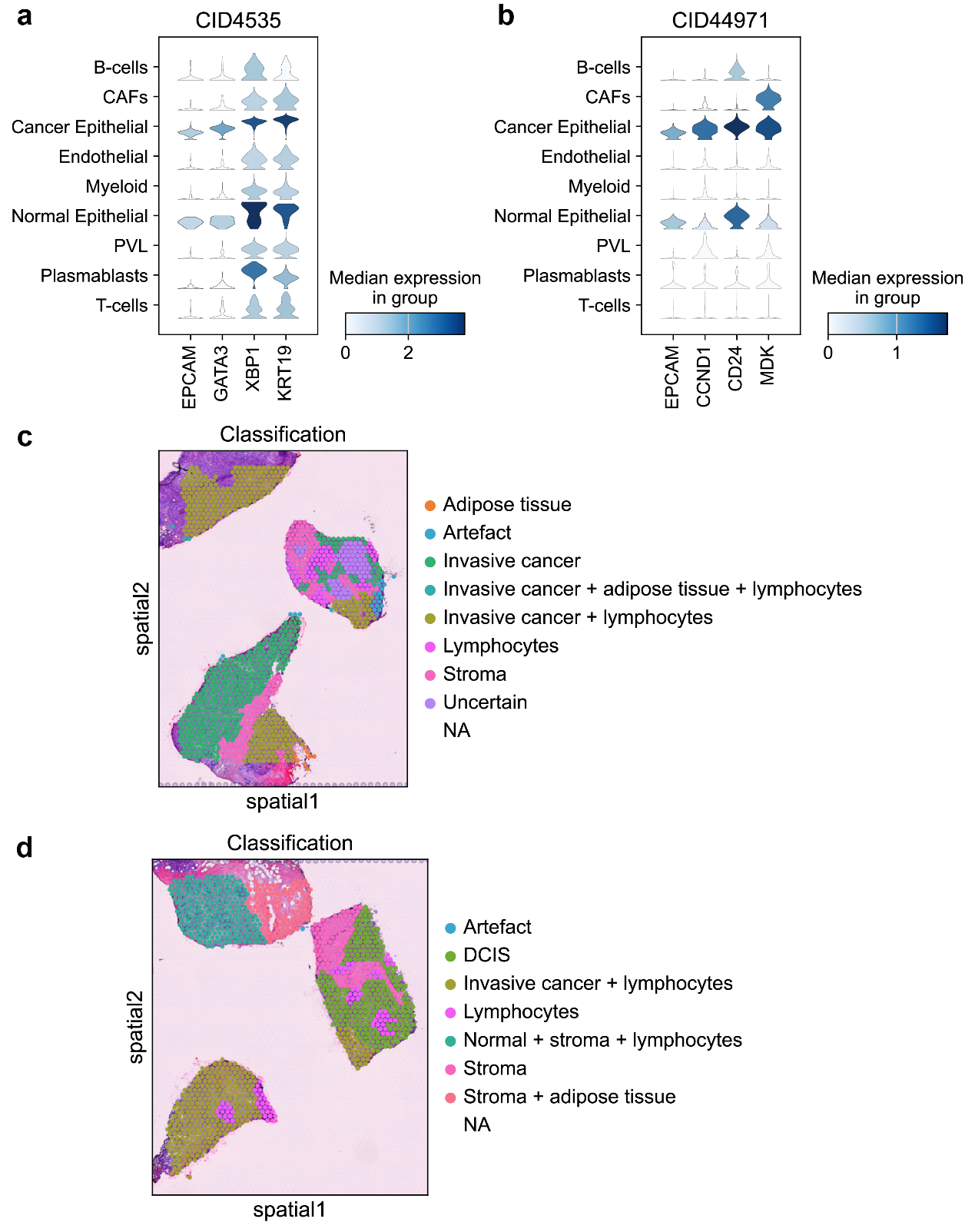


**Supplementary Fig. 13 | Tumor epithelial DEGs and tissue annotations in breast cancer samples.** **a**, Violin plots showing tumor epithelial–specific DEGs in CID4535. **b**, Violin plots showing tumor epithelial–specific DEGs in CID44971. **c**, Tissue region annotations in CID4535 from the original study. **d**, Tissue region annotations in CID44971 from the original study.


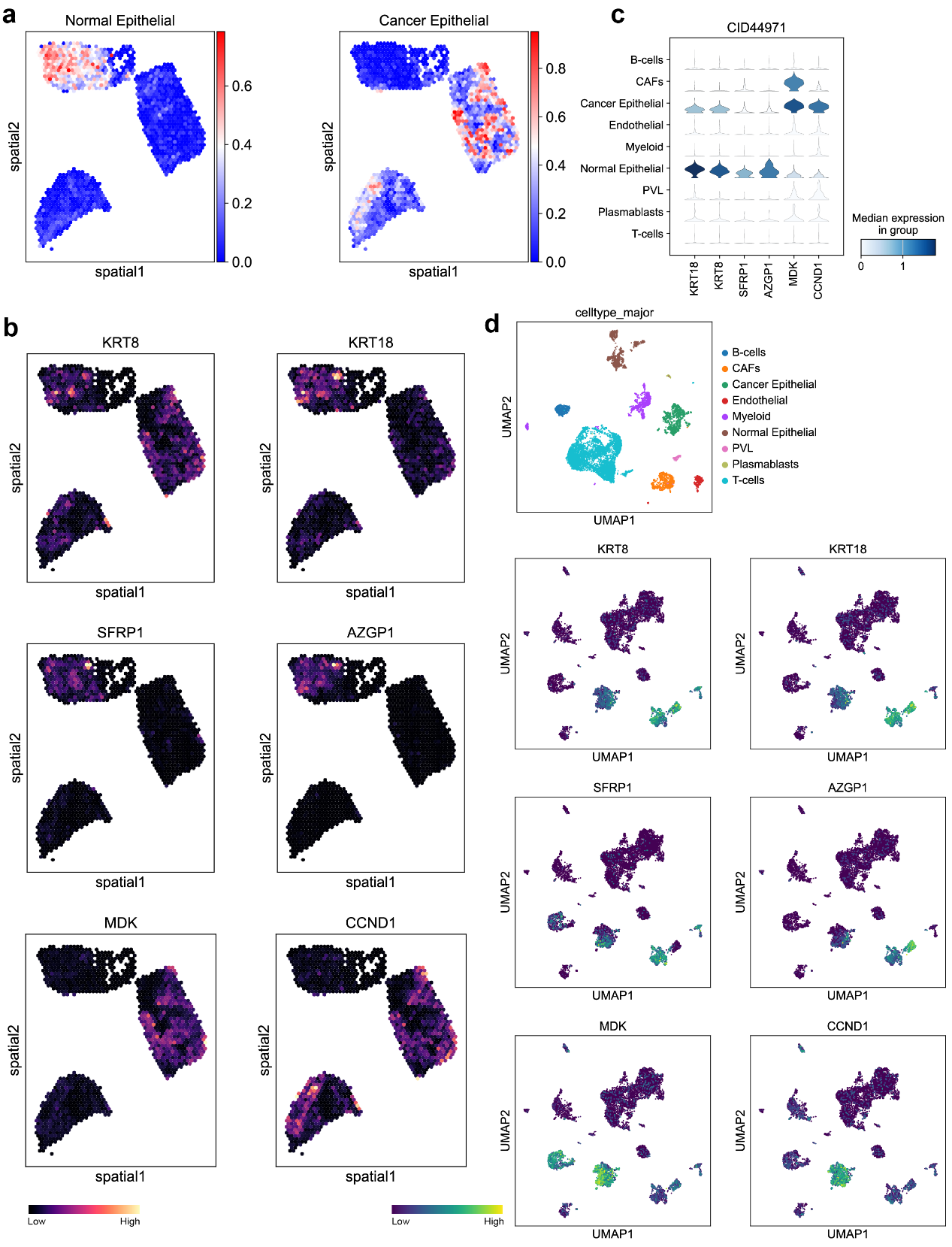


**Supplementary Fig. 14 | Spatial and transcriptional characterization of normal and cancer epithelial cell states in breast cancer tissue.** **a**, Spatial distributions of normal epithelial and cancer epithelial cells predicted by STAID in the breast cancer sample CID44971. **b**, Spatial expression patterns of selected genes: KRT8 and KRT18 for both cell types; SFRP1 and AZGP1 for normal epithelial cells; MDK and CCND1 for cancer epithelial cells. **c**, Violin plots showing median expression levels of the above genes. **d**, UMAP visualization of the scRNA-seq reference dataset (top panel) and gene expression levels of the selected genes.


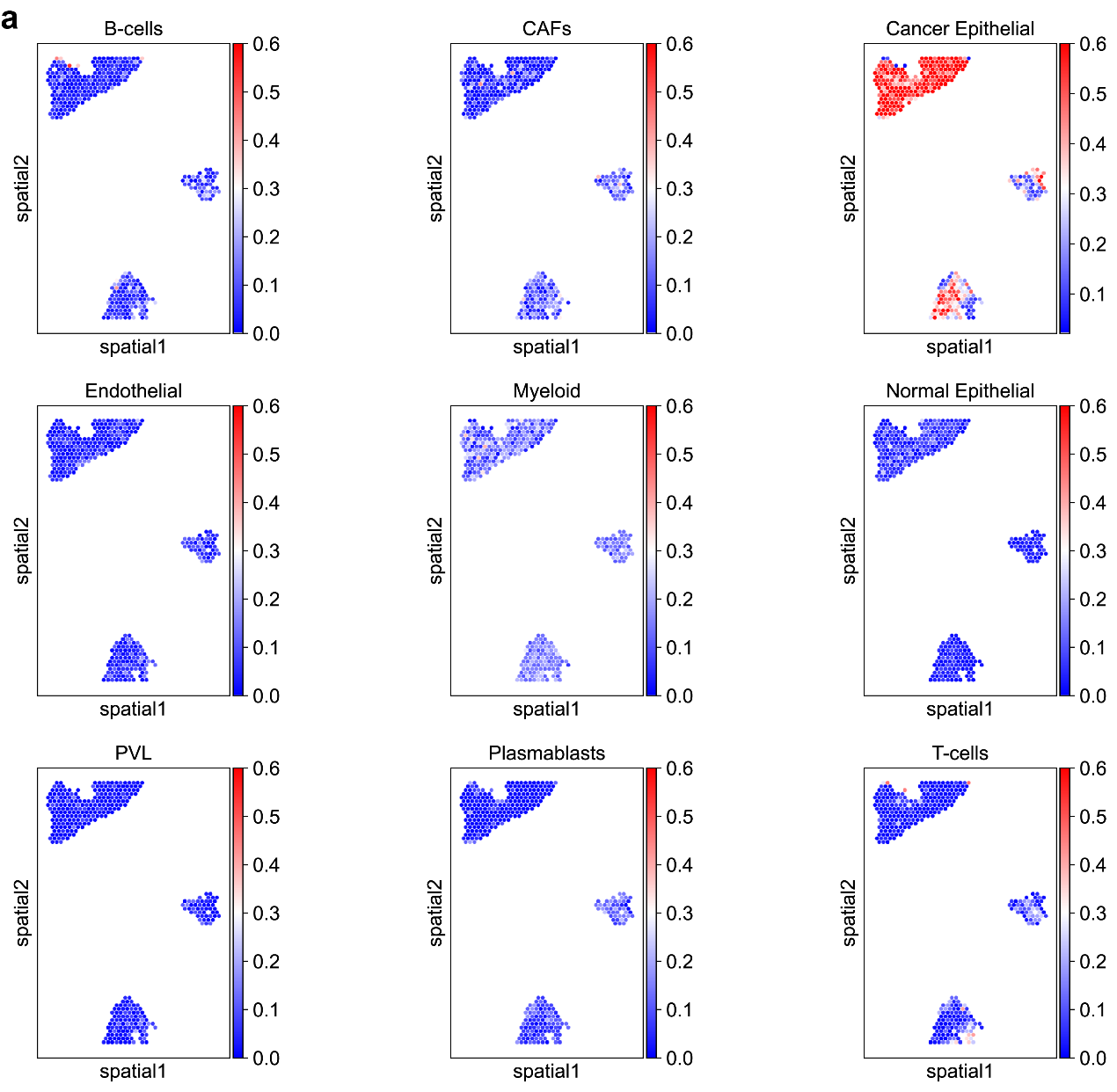


**Supplementary Fig. 15 | Cell-type distributions within the tissue section of CID4535. a**, Spatial distributions of cell types within the “invasive tumor + lymphocytes” region in CID4535.


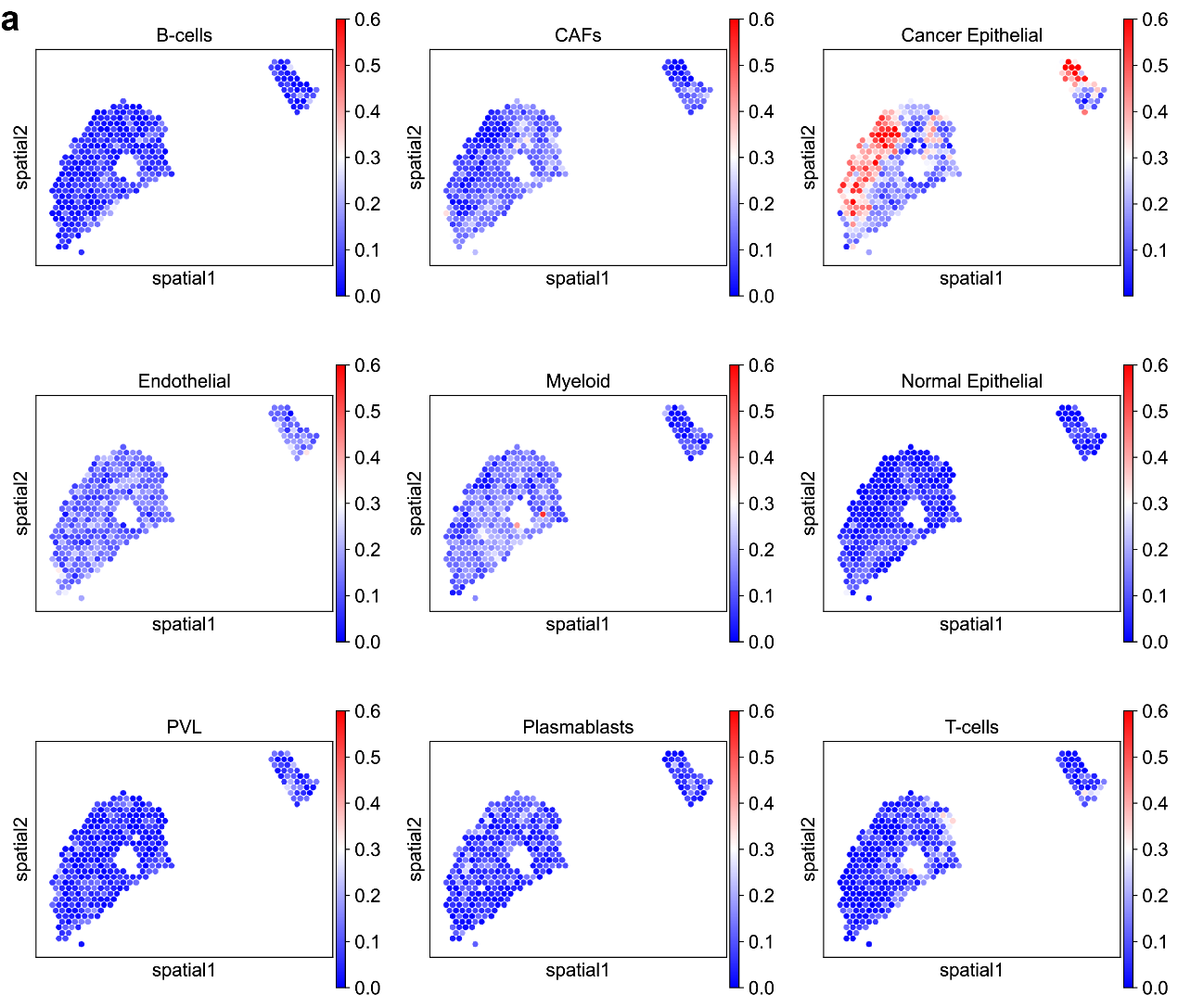


**Supplementary Fig. 16 | Cell-type distributions within the tissue section of CID44971. a**, Spatial distributions of cell types within the “invasive tumor + lymphocytes” region in CID44971.


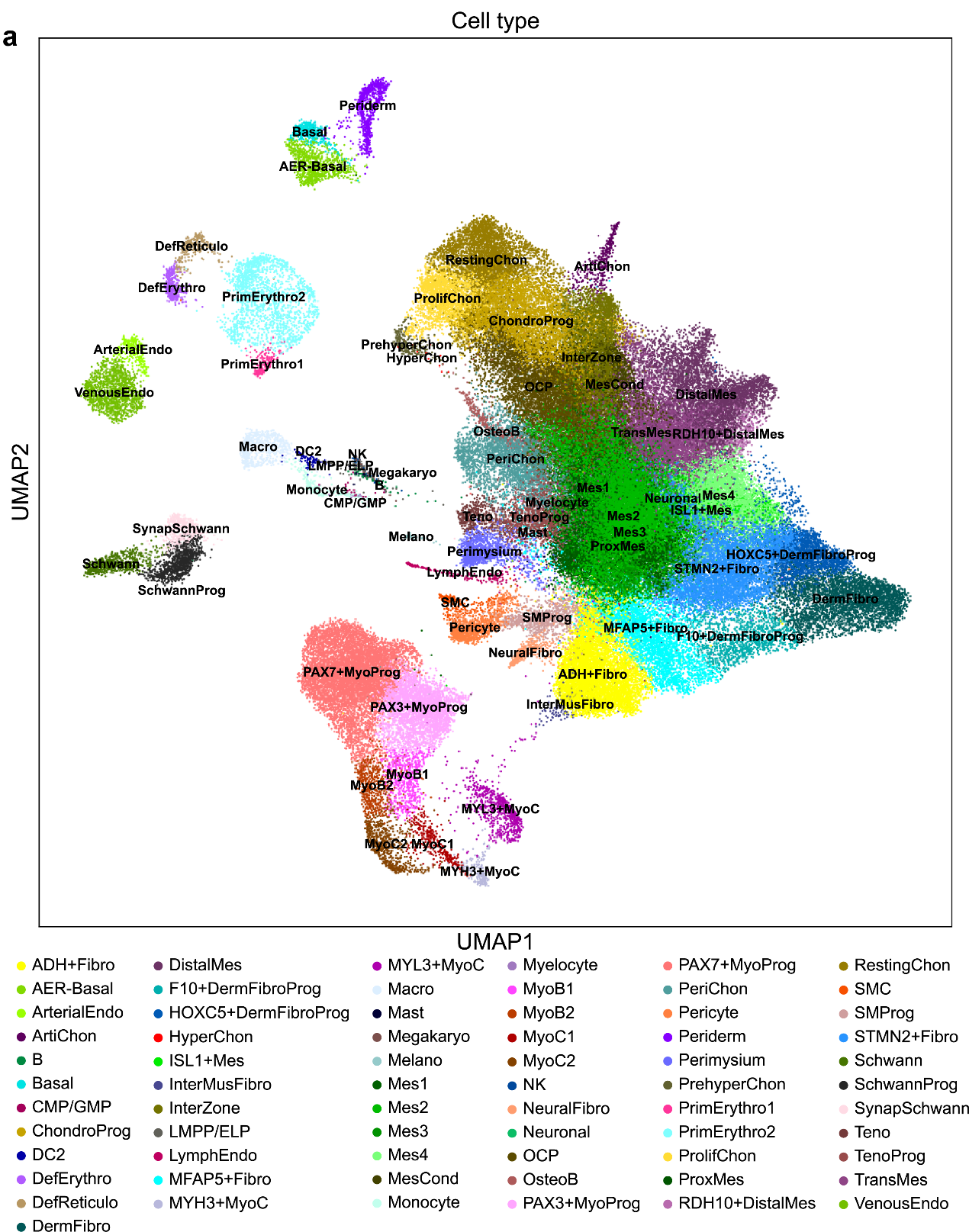


**Supplementary Fig. 17 | scRNA-seq annotation of human embryonic limb cells. a**, UMAP visualization of single cells from the reference scRNA-seq dataset, colored by annotated cell types as reported in the original study.

**
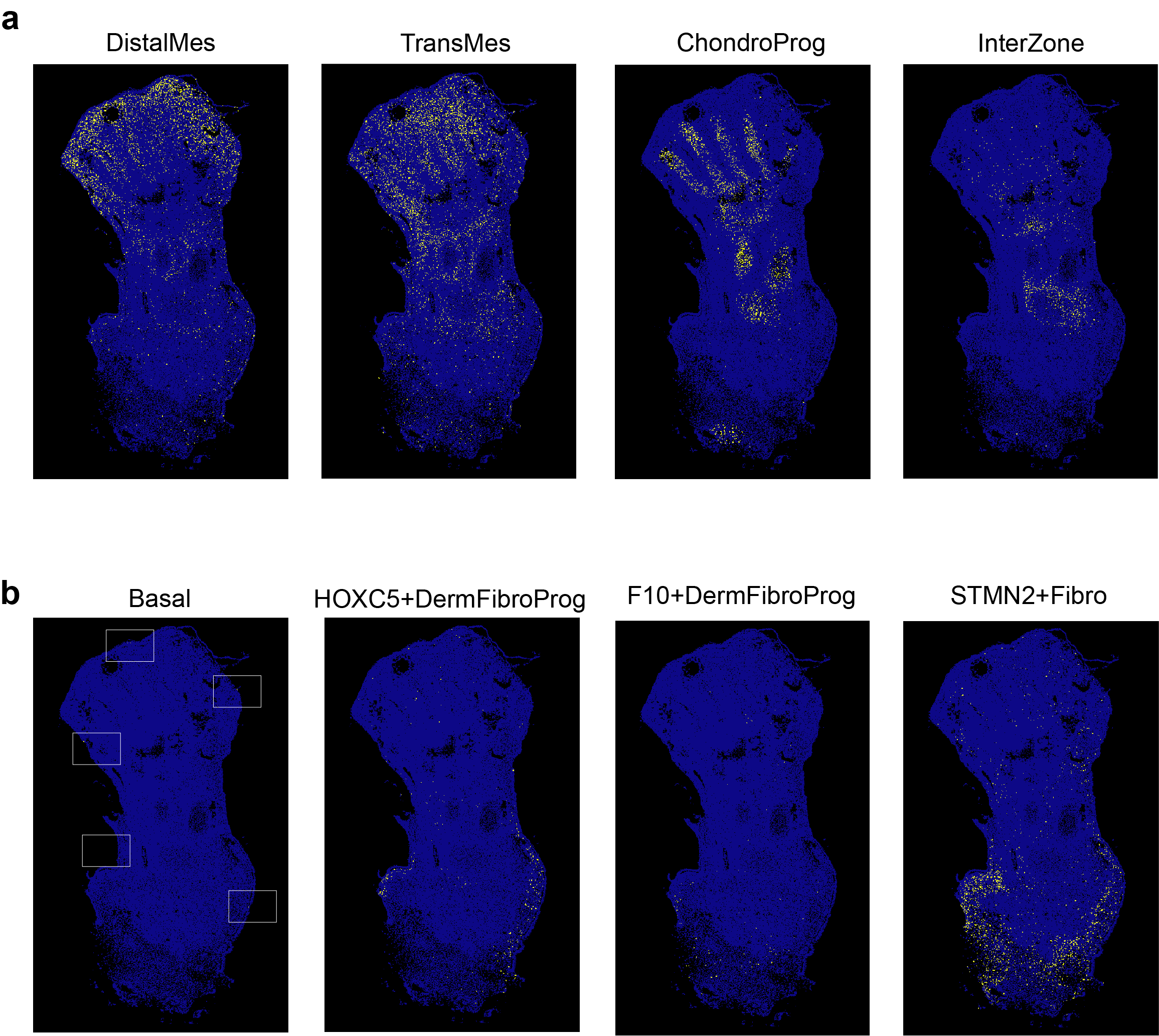
**

**Supplementary Fig. 18 | Cell-type distribution in independent ISS data during embryonic limb development. a**, Spatial distributions of four cell types: DistMes, TransMes, ChondroProg, and InterZone. Yellow dots indicate cells of the shown type, blue dots indicate other cell types, and black represents background. **b**, Spatial distributions of four additional cell types: Basal, HOXC5+ Dermal Fibroblast Progenitors (HOXC5+DermFibroProg), F10+ Dermal Fibroblast Progenitors (F10+DermFibroProg), and STMN2+ Fibroblasts (STMN2+Fibro). Color coding is the same as in panel (a).


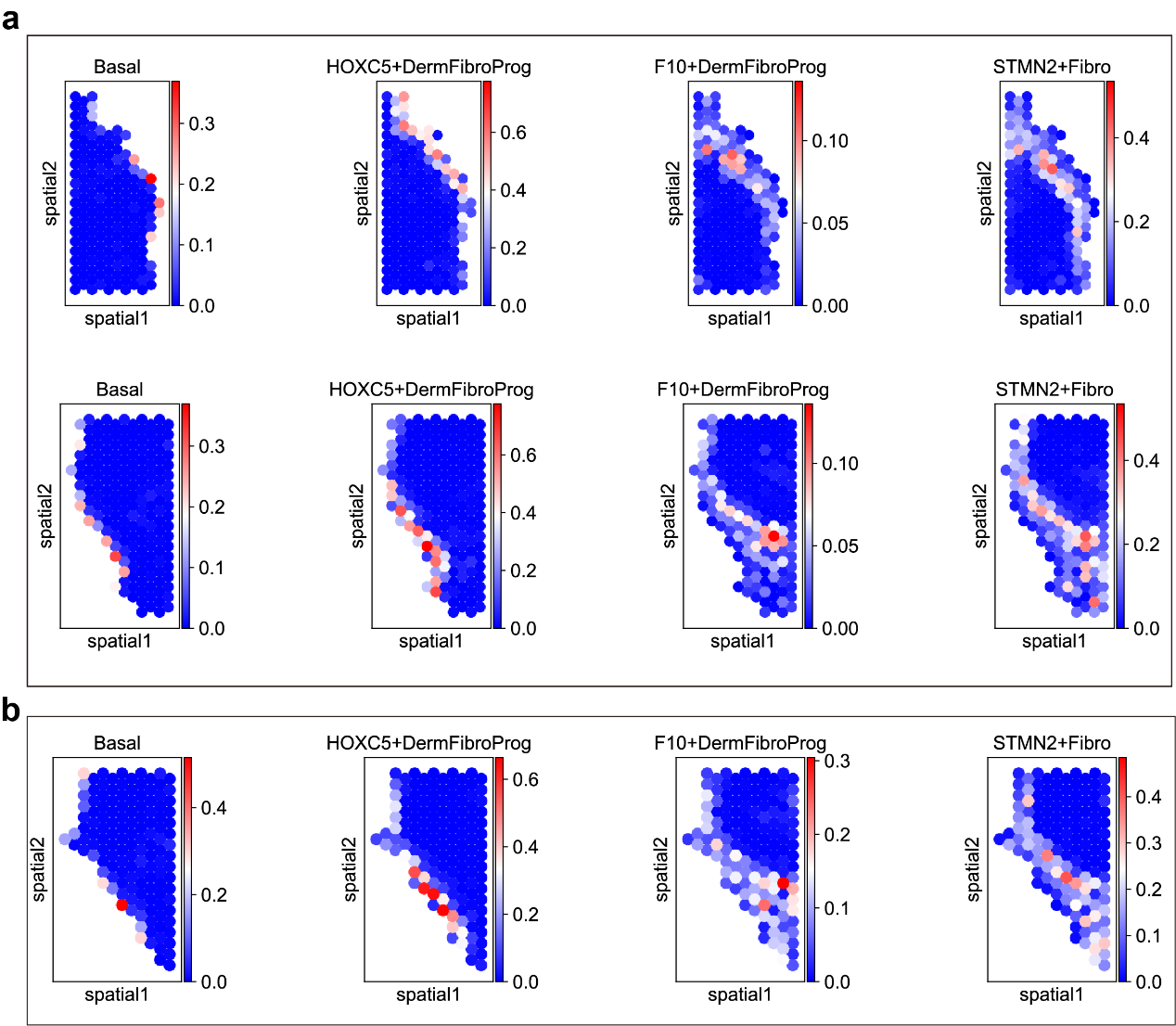


**Supplementary Fig. 19 |** **Cell-type distribution patterns within the selected tissue regions of human embryonic limb development datasets.** **a**, Spatial distributions of four fibroblast-related cell types in the 6.2 PCW: Basal, HOXC5+DermFibroProg, F10+DermFibroProg, and STMN2+Fibro. **b**, Spatial distributions of four fibroblast-related cell types in the 5.6 PCW: Basal, HOXC5+DermFibroProg, F10+DermFibroProg, and STMN2+Fibro.

**
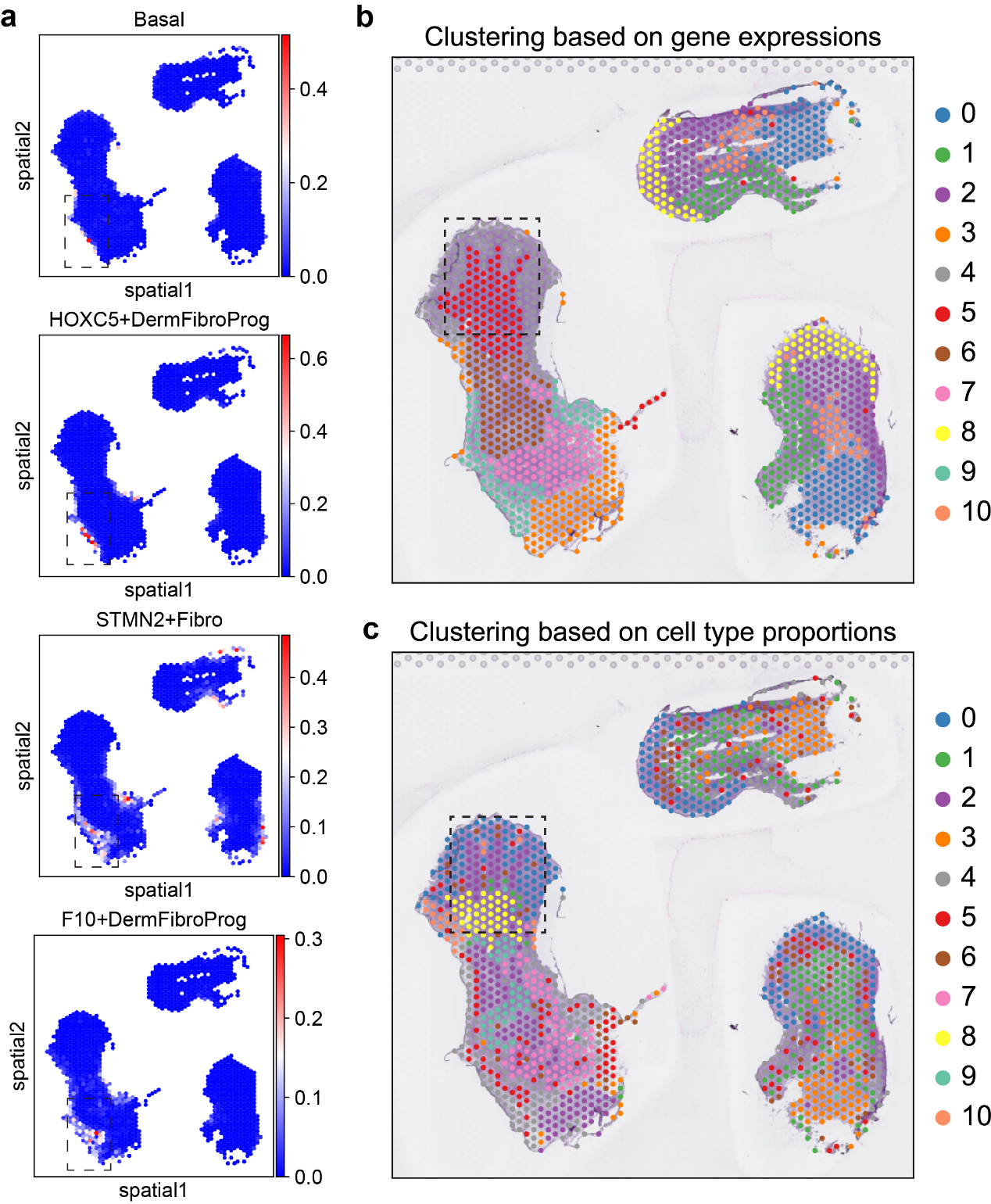
**

**Supplementary Fig. 20 | Spatial mapping and clustering of human embryonic limb at 5.6 PCW. a**, Spatial distributions of four cell types, Basal, HOXC5+ Dermal Fibroblast Progenitors (HOXC5+DermFibroProg), F10+ Dermal Fibroblast Progenitors (F10+DermFibroProg), and STMN2+ Fibroblasts (STMN2+Fibro), as inferred by STAID. **b–c**, Clustering of spots based on gene expression (b) or STAID-derived cell type proportions (c). Clustering using STAID-derived cell-type compositions more accurately delineates tissue structures compared with clustering based on gene expression alone.


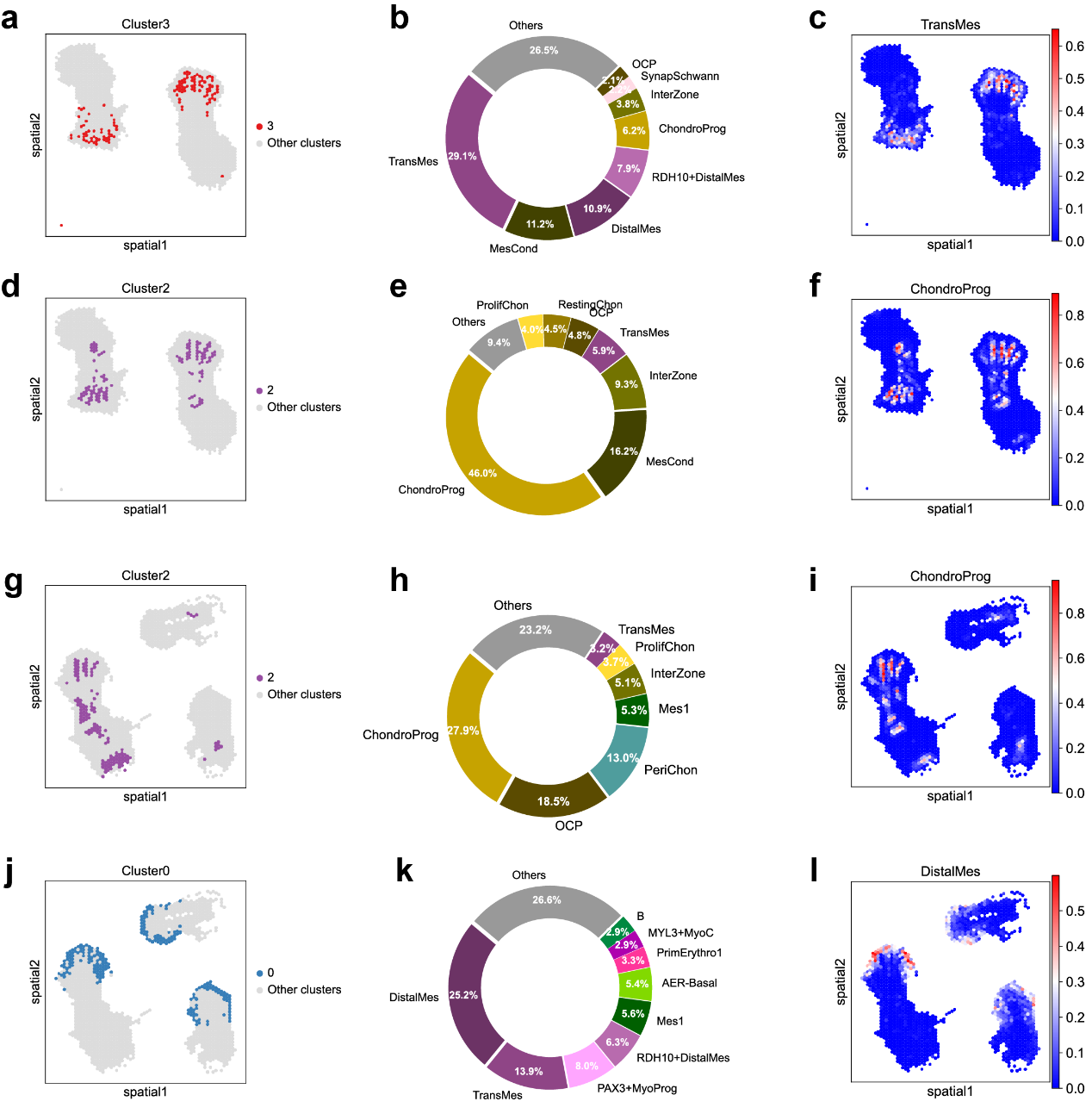
**Supplementary Fig. 21 | Cell type information of some clusters of human embryonic limb development data. a**, Spatial distribution of cluster3 at 6.2 PCW. **b**, Cell-type composition in cluster 3. **c**, Spatial expression pattern of TransMes, which is the dominant cell type in cluster 3. **d**, Spatial distribution of cluster 2 at 6.2 PCW. **e**, Cell-type composition in cluster 2. **f**, Spatial expression pattern of ChondroProg, which is the dominant cell type in cluster 2. **g**, Spatial distribution of cluster 2 at 5.6 PCW. **h**, Cell-type composition in cluster 2. **i**, Spatial expression pattern of ChondroProg, which is the dominant cell type in cluster 2. **j**, Spatial distribution of cluster 0 at 5.6 PCW. **k**, Cell-type composition in cluster 0. **l**, Spatial expression pattern of DistalMes, which is the dominant cell type in cluster 0.


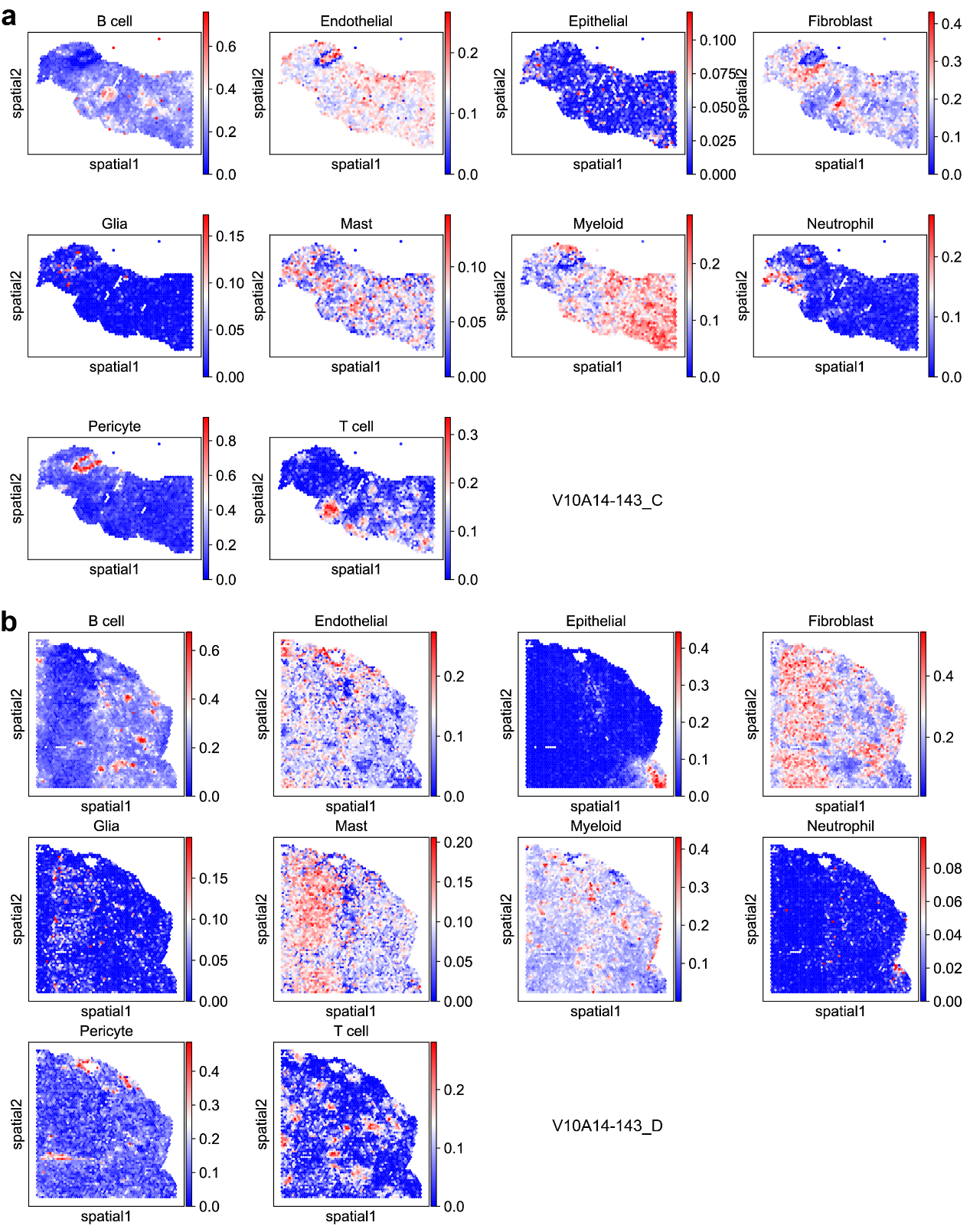


**Supplementary Fig. 22 | Spatial distributions of cell types in two stricture sections infered by STAID.** **a**, Spatial patterns of cell types of V10A14-143_C inferred by STAID. **b**, Spatial patterns of cell types of V10A14-143_D inferred by STAID.


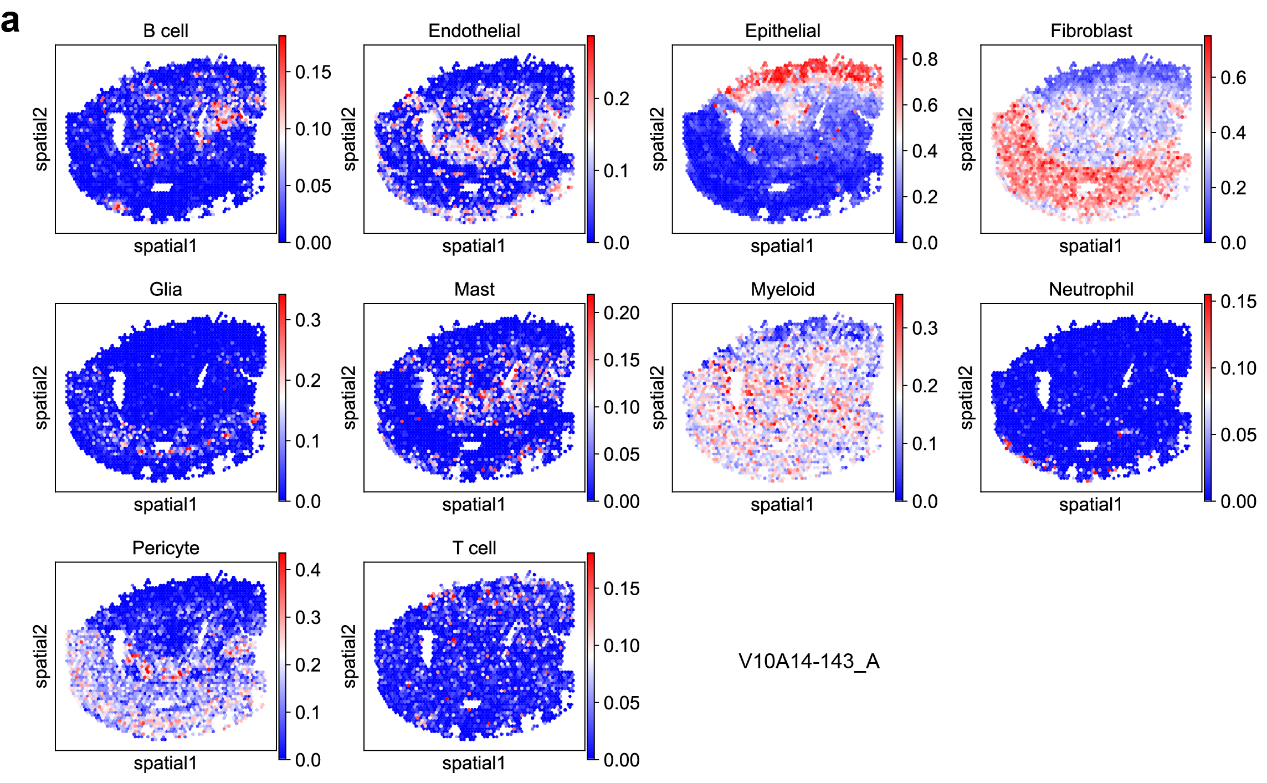


**Supplementary Fig. 23 | Spatial distributions of cell types in the adjacent non-stricture section infered by STAID.** **a**, Spatial patterns of cell types of V10A14-143_A inferred by STAID.

**
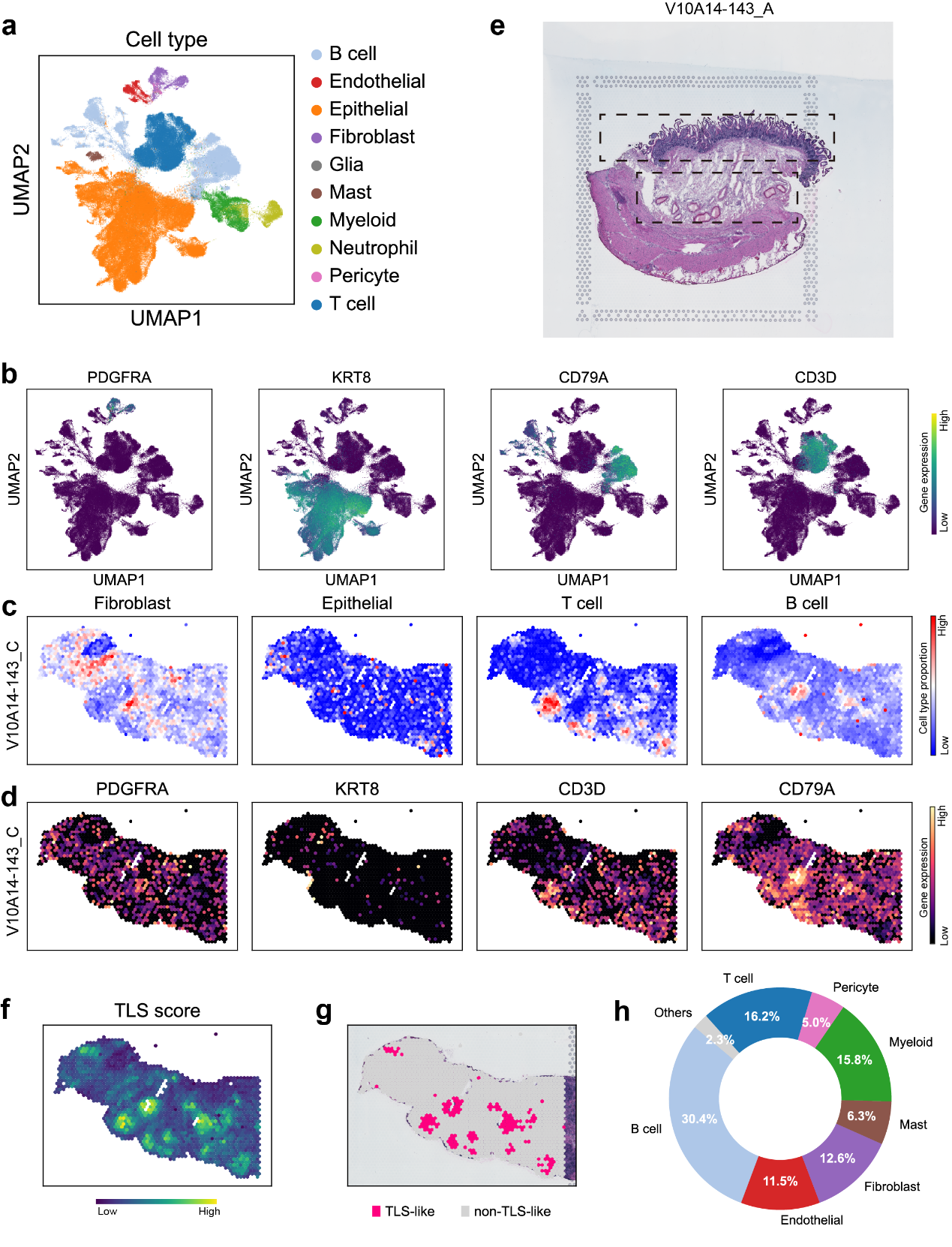
**

**Supplementary Fig. 24 | STAID deconvolution of spatial cell-type distributions and TLS-like niches in Crohn’s disease intestinal tissue. a**, UMAP visualization of the scRNA-seq reference dataset used for STAID deconvolution, colored by annotated major cell lineages. **b**, UMAP plots of marker genes *PDGFRA*, *KRT8*, *CD79A* and *CD3D* in the scRNA-seq reference **c**, Spatial distributions of fibroblasts, epithelial cells, T cells and B cells V10A14-143_C predicted by STAID. **d**, Spatial expression patterns of marker genes in V10A14-143_C. **e**, Hematoxylin and eosin (H&E) staining of non-stricture sample V10A14-143_A. The top box indicates the luminal surface, and the bottom indicates Subepithelial lamina propria. **f**, Spatial map of TLS scores in V10A14-143_C, defined as the mean log-expression of TLS signature genes. **g**, Spatial annotation of TLS-like enriched and non-TLS-like niches in V10A14-143_C based on TLS score thresholding. **h**, Quantified cell-type proportions within TLS regions in V10A14-143_C derived from STAID predictions.

**Supplementary Tables**

**Supplementary Table 1. P-values of four metrics comparing STAID with second-best method**

| Metric | Dataset | Level | Best method | Second-best method | P-value (Wilcoxon) | Significance |
| --- | --- | --- | --- | --- | --- | --- |
| MAE | Scenario 1 | spot-level | STAID | SONAR | 1.40E-282 | *** |
|  | Scenario 1 | cell-type-level | STAID | SONAR | 6.10E-05 | *** |
|  | Scenario 2 | spot-level | STAID | SONAR | 3.95E-71 | *** |
|  | Scenario 2 | cell-type-level | STAID | SONAR | 0.004180908 | ** |
|  | Scenario 3 | spot-level | STAID | SONAR | 4.67E-268 | *** |
|  | Scenario 3 | cell-type-level | STAID | SONAR | 0.000183105 | *** |
|  | Scenario 4 | spot-level | STAID | SONAR | 9.88E-297 | *** |
|  | Scenario 4 | cell-type-level | STAID | SONAR | 9.16E-05 | *** |
|  | Scenario 5 | spot-level | STAID | SONAR | 1.53E-120 | *** |
|  | Scenario 5 | cell-type-level | STAID | SONAR | 0.001678467 | ** |
| JSD | Scenario 1 | spot-level | STAID | SONAR | 0 | *** |
|  | Scenario 1 | cell-type-level | STAID | SONAR | 3.05E-05 | *** |
|  | Scenario 2 | spot-level | STAID | SONAR | 2.41E-92 | *** |
|  | Scenario 2 | cell-type-level | STAID | SONAR | 0.32251 | ns |
|  | Scenario 3 | spot-level | STAID | SONAR | 0 | *** |
|  | Scenario 3 | cell-type-level | STAID | SONAR | 6.10E-05 | *** |
|  | Scenario 4 | spot-level | STAID | SONAR | 0 | *** |
|  | Scenario 4 | cell-type-level | STAID | SONAR | 3.05E-05 | *** |
|  | Scenario 5 | spot-level | STAID | SONAR | 4.75E-127 | *** |
|  | Scenario 5 | cell-type-level | STAID | SpatialDWLS | 0.159058 | ns |
| RMSE | Scenario 1 | spot-level | STAID | SONAR | 1.24E-198 | *** |
|  | Scenario 1 | cell-type-level | STAID | SONAR | 0.000305176 | *** |
|  | Scenario 2 | spot-level | STAID | SONAR | 2.98E-74 | *** |
|  | Scenario 2 | cell-type-level | STAID | SONAR | 0.005157471 | ** |
|  | Scenario 3 | spot-level | STAID | SONAR | 3.53E-200 | *** |
|  | Scenario 3 | cell-type-level | STAID | SONAR | 0.000305176 | *** |
|  | Scenario 4 | spot-level | STAID | SONAR | 2.00E-213 | *** |
|  | Scenario 4 | cell-type-level | STAID | SONAR | 0.000152588 | *** |
|  | Scenario 5 | spot-level | STAID | SONAR | 8.53E-98 | *** |
|  | Scenario 5 | cell-type-level | STAID | SONAR | 0.005157471 | ** |
| PCC | Scenario 1 | spot-level | STAID | SONAR | 1.75E-174 | *** |
|  | Scenario 1 | cell-type-level | STAID | Stereoscope | 3.05E-05 | *** |
|  | Scenario 2 | spot-level | STAID | RCTD | 6.09E-65 | *** |
|  | Scenario 2 | cell-type-level | STAID | Stereoscope | 0.044311523 | * |
|  | Scenario 3 | spot-level | STAID | SONAR | 7.08E-159 | *** |
|  | Scenario 3 | cell-type-level | STAID | Stereoscope | 6.10E-05 | *** |
|  | Scenario 4 | spot-level | STAID | SONAR | 9.34E-180 | *** |
|  | Scenario 4 | cell-type-level | STAID | Stereoscope | 3.05E-05 | *** |
|  | Scenario 5 | spot-level | STAID | SONAR | 7.07E-69 | *** |
|  | Scenario 5 | cell-type-level | STAID | Stereoscope | 0.006286621 | ** |

ns: not significant; two-sided Wilcoxon signed-rank test

**Supplementary Table 2. P-values of four metrics comparing STAID with second-best method in MERFISH.**

| Metric | Dataset | Level | Best method | Second-best method | P-value (Wilcoxon) | Significance |
| --- | --- | --- | --- | --- | --- | --- |
| MAE | MERFISH-simulation | spot-level | STAID | RCTD | 9.04E-40 | *** |
|  | MERFISH-simulation | cell-type-level | STAID | RCTD | 0.02734375 | * |
| JSD | MERFISH-simulation | spot-level | STAID | RCTD | 4.54E-54 | *** |
|  | MERFISH-simulation | cell-type-level | STAID | Cell2location | 0.001953125 | ** |
| RMSE | MERFISH-simulation | spot-level | STAID | RCTD | 3.64E-43 | *** |
|  | MERFISH-simulation | cell-type-level | STAID | RCTD | 0.013671875 | * |
| PCC | MERFISH-simulation | spot-level | STAID | RCTD | 8.18E-32 | *** |
|  | MERFISH-simulation | cell-type-level | STAID | RCTD | 0.037109375 | * |

ns: not significant; two-sided Wilcoxon signed-rank test

**Supplementary Table 3. P-values of four metrics comparing STAID with second-best method in SeekSpace.**

| Metric | Dataset | Level | Best method | Second-best method | P-value (Wilcoxon) | Significance |
| --- | --- | --- | --- | --- | --- | --- |
| MAE | SeekSpace-simulation | spot-level | STAID | Cell2location | 1.91E-50 | *** |
|  | SeekSpace-simulation | cell-type-level | STAID | Cell2location | 0.013671875 | * |
| JSD | SeekSpace-simulation | spot-level | STAID | Cell2location | 1.01E-38 | *** |
|  | SeekSpace-simulation | cell-type-level | STAID | Cell2location | 0.921875 | ns |
| RMSE | SeekSpace-simulation | spot-level | STAID | Cell2location | 1.58E-44 | *** |
|  | SeekSpace-simulation | cell-type-level | STAID | Cell2location | 0.009766 | ** |
| PCC | SeekSpace-simulation | spot-level | STAID | Cell2location | 4.24E-27 | *** |
|  | SeekSpace-simulation | cell-type-level | STAID | RCTD | 0.130859 | ns |

ns: not significant; two-sided Wilcoxon signed-rank test

**Supplementary Table S4. TLS marker genes.**

| TLS markers |
| --- |
| CD4 |
| CD8A |
| CD74 |
| CD79A |
| IL7R |
| ITGAE |
| CD1D |
| CD3D |
| CD3E |
| CD8B |
| CD19 |
| CD22 |
| CD52 |
| CD79B |
| CR2 |
| CXCL13 |
| CXCR5 |
| FCER2 |
| MS4A1 |
| PDCD1 |
| PTGDS |
| TRBC2 |
